# Improving adhesion and targeting of adherent areas for high-throughput cryo electron tomography of vitreous sections

**DOI:** 10.1016/j.yjsbx.2026.100167

**Published:** 2026-10-20

**Authors:** Fatima Taiki, Helmut Gnaegi, Diana Grewe, Nils Marechal, Mikhail Eltsov, Amélie Leforestier

**Affiliations:** a Laboratoire de Physique des Solides (LPS), CNRS, Université Paris-Saclay Orsay, France; b Diatome Ltd, Helmstrasse 1, Nidau, Switzerland; c Buchmann Institute for Molecular Life Sciences, Goethe University, Frankfurt am Main, Germany; d Centre for Integrative Biology (CBI), Department of Integrated Structural Biology, IGBMC, CNRS, Inserm, Université de Strasbourg, Illkirch, France

**Keywords:** Cryo electron tomography, Vitreous cryo-sections, CEMOVIS, Cryo electron microscopy, Ultra cryo-microtomy

## Abstract

Cellular cryo electron tomography explores tissue and cells in their unstained flash-frozen native state, revealing in situ the structure of macromolecules together with their local environment and interactions with partners, also known as molecular sociology. To obtain thin samples, cryo-FIB milling is nowadays the most popular method, with impressive successes. The alternative, cryo-ultramicrotomy, is often overlooked on account of poorly reproducible attachment of cryo-sections to their support, resulting in low throughput. We optimized the workflow, focusing on section adhesion and their support grids. We thus increased cryo electron tomography of vitreous section throughput, to equal that of thin film, typically leading to several tens to hundreds of cryo-tomograms per ribbon of sections. This opens the way to new advances in cellular cryo electron tomography, as the method is devoid of ion beam damage, and can provide large surfaces and serial sections of any type of sample from cells to tissues, organs or even entire organisms. In addition, section thickness can be tuned down to 30–50 nm, which may be an advantage for imaging small molecular complexes, such as DNA and nucleosomes.

## Highlights

• adhesion of cryo-sections on carbon films is monitored at a glance at macroscopic and microscopic levels.
• cryo-section adhesion on carbon film propagates upon two-step electrostatic charging with an ionizer.
• different types of support grids are tested for high throughput 2D and 3D imaging of vitreous cryo-sections.

## Graphical abstract

**Figure:**
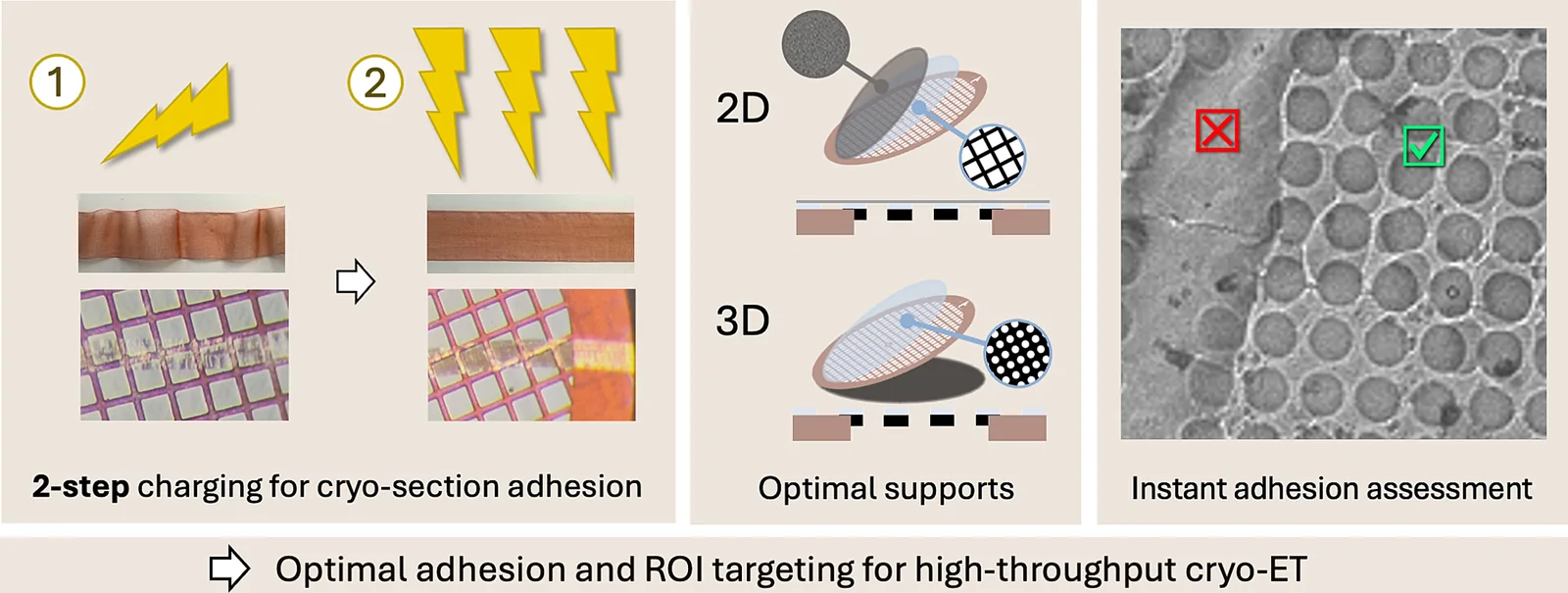

## 1 Introduction

Cellular cryo electron tomography (cryo-ET) is experiencing continuous spectacular developments, since its opening of the field of molecular sociology (Robinson et al., 2007), revealing with increasing resolution and complexity the structures and interactions of macromolecules at work inside the cell (Nogales and Mahamid, 2024). These achievements have been possible thanks to new generations of high-end stable and automated electron microscopes and direct electron detectors, and the development of cryo-FIB milling to achieve sample thinning to electron transparency, i.e. less than a few hundred nanometers, and ideally below 200 nm (Villa et al., 2013) (Rigort and Plitzko, 2015). The historic alternative to cryo-FIB milling is cryo-sectioning – also known as CEMOVIS (cryo electron microscopy of vitreous sections) – which cuts samples into thin slices with a diamond knife (Al Amoudi et al., 2004). CEMOVIS offers a number of advantages. (i) It is able to provide ultra-thin samples, with sections as thin as 20 nm (Ng et al., 2020), and more routinely in the 40–80 nm range, whereas cryo-FIB lamellae are usually thicker than 100 nm, routinely in the 150–200 nm range. This may be an advantage when high resolution is needed or to image small complexes and molecules, such as DNA (Fatmaoui et al., 2026). (ii) Cryo-sectioning provides access to large, entire volumetric domains of a specimen. Not only the typical section area is about 20 times larger than a cryo-FIB lamella, but cryo-sectioning generates serial sections of a sample, whereas FIB-milling destroys most of it, keeping only one lamella per milled object – or a few sparse ones for the Serial lift-out technique (Schiøtz et al., 2024). In addition, cryo-sectioning can be applied equivalently to any type of sample, cell suspensions or tissues, after high pressure freezing. In contrast, if on-grid cryo-FIB milling of cells that can be vitrified by plunge freezing is now a high throughput technique, it is much more challenging for tissues, for which complex lift out methodologies are required (Parmenter and Nizamudeen, 2021). Lastly, cryo-FIB milling is associated to ion beam damage that can extend to a depth of 60 nm from the surface, limiting the recovery of information therein (Lucas and Grigorieff, 2023), which is not the case with cryo-sectioning (Elferich et al., 2025) (Al-Amoudi et al., 2025). A few studies have indeed highlighted CEMOVIS potential for high resolution imaging, with structural information retrieved down to 2.9 Å (Moriscot et al., 2023) or 7.9 Å (Sader et al., 2009), or 3D structure reconstruction at sub-nanometer resolution, down to 3.5 Å and even 3 Å on ribosomal subunits (Elferich et al., 2025)(Al Amoudi et al., 2025), very similar to what can be obtained from FIB-milled samples (Hoffmann et al., 2022). However, CEMOVIS is nowadays overlooked, on account of the compression artefacts induced by the cutting process (Al Amoudi et al., 2005), but also because of the low throughput of the method, especially for the recording of tilt series for cryo-tomographic reconstructions. The poor yield in recording stable tilt series on cryo-sections is primarily due to poor and irreproducible adhesion of cryo-sections to their support grid, but also to the difficulty in assessing perfect adhesion a priori, before the recording of the tilt series themselves. In addition, for cryo-ET imaging in high end microscopes, grids must be clipped, which can result in breaks and loss of sample areas. This is rarely a problem for plunge-frozen samples whose support film is reinforced by its covering with the layer of frozen buffer solution, but becomes an issue when working with cryo-sections simply deposited on a thin support film.

In this study, we propose an optimized cryo-sectioning workflow leading to high throughput cryo-ET recording. We introduce an extra step to improve adhesion, using the electrostatic press developed for section attachment by Pierson et al. (2010), away from the knife edge. We also propose a global real-time monitoring test of adhesion upon section collection on grids, and a local adhesion characterization in low magnification mode before image/tilt series recording. Lastly, we test a variety of support commercial and home-modified grids, and determine optimal supports for either 2-dimensional (2D) or 3-dimensional (3D) tomographic imaging.

## 2 Material and methods

### 2.1 Sample vitrification

As previously described (Eltsov et al., 2018; Fatmaoui et al., 2026), stage 13–15 Drosophila embryos were dechorionated in 50% (v:v) bleach, rinsed in PBS and transferred into a Leica type A gold-plated copper carrier. The carrier was then filled with 25% (w:w) dextran (40 kDa, Sigma-Aldrich) and 0.25% of NP-40 (Sigma-Aldrich) in PBS and covered with the flat side of a Leica type B aluminum carrier. The addition of NP-40 surfactant is needed to embed the hydrophobic vitelline membrane of the embryo in the Dextran solution, which is used here as a convenient embedding medium and does not exert any osmotic effect and is easy to section. The sandwich was transferred into a Baltec HPM010 freezing machine, and vitrified with liquid nitrogen at 2045 bar.

### 2.2 Cryo-sectioning

High-pressure frozen samples were transferred into the chamber of a FC6/UC6 cryo-microtome (Leica Microsystems) installed in a controlled environment room, with the relative humidity set to 20–25% and the temperature to 23 °C. The cryochamber of the microtome was cooled down to −140 °C. The aluminum cover was removed and the gold-plated copper carrier filled with the sample mounted inside a flat sample holder. A diamond trimming tool trim 45 (Diatome) was used to remove the metal edges of the carrier, and a ~ 50 μm × 70 μm × 50 μm pyramid was trimmed within the central nervous system of the embryo. Final trimming steps were realized using a trim 20 (Diatome). Cryo-sections were cut from the pyramid at a nominal feed of 50 or 75 nm with a cryo 25° diamond knife (Diatome) at a cutting speed of 0.4 mm/s. A Leica EM Crion tunable antistatic device in discharge mode was used to optimize section generation and gliding along the knife. Ribbons of serial sections were pulled and collected using a double micromanipulator (Leica), which lets one control the generation of millimeter-long ribbons of sections (Pierson et al., 2010) (Studer et al., 2014). The sections were then collected onto support grids. Adhesion is obtained using the Leica EM Crion antistatic device used in charging mode, which acts as an electrostatic press (Pierson et al., 2010): a first pulse is applied just after ribbon collection on the grid (Fig. 1B, C). For some samples, the grid was moved away from the knife using the micromanipulator, then rotated to be oriented perpendicular to the electrode, and submitted to a second charging pulse (Fig. 1C'). Both charging pulses are applied until no further macroscopic contrast change is observed (maximal adhesion, see Supplementary movie), i.e. 1 to 3 s for the first charging step, and 3 to 10 s for the second charging step (see *Results*). Applying one long 5 to 10 s pulse or 5 to 10 short (1 s) ones does not make any difference.

**Fig. 1:**
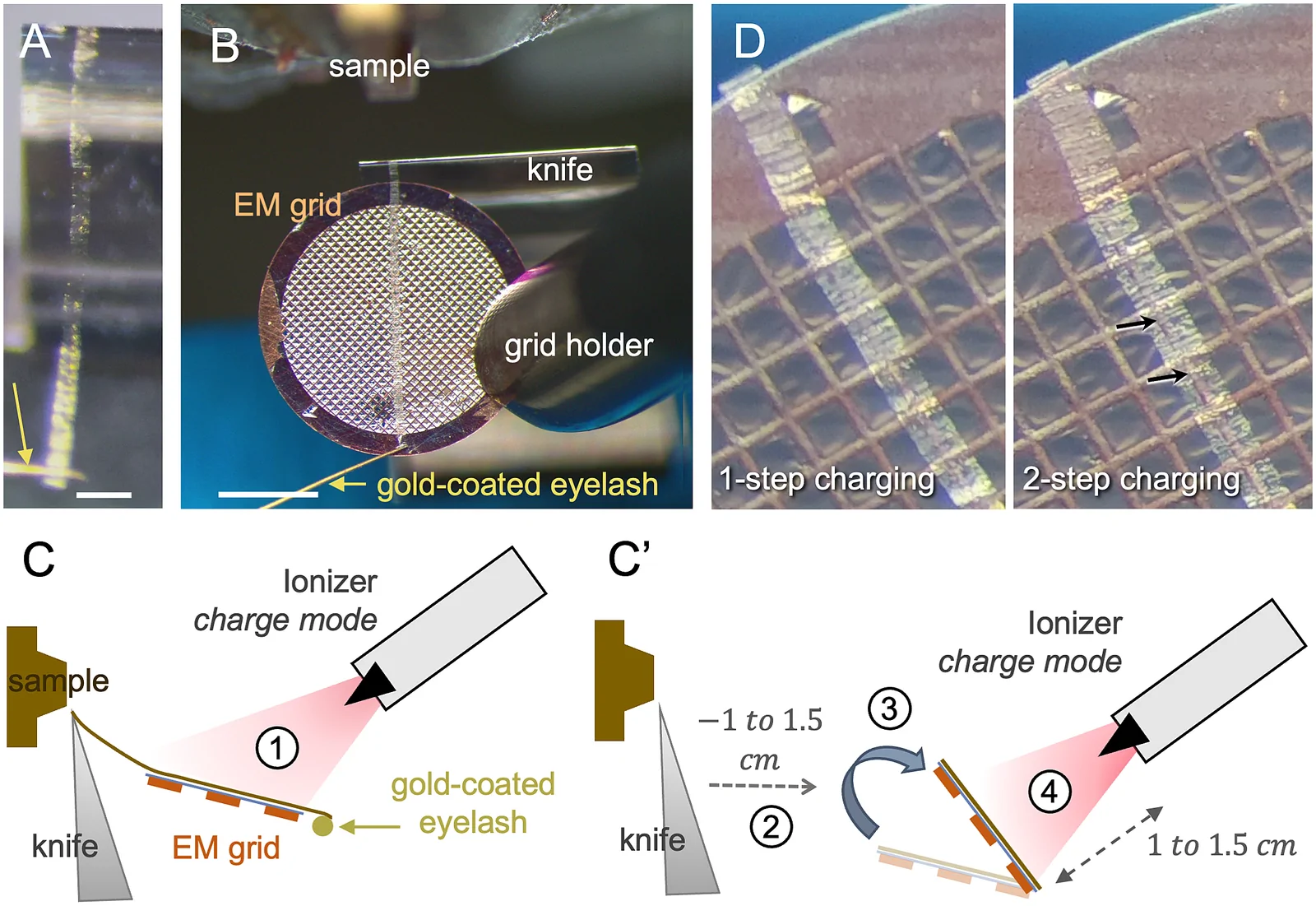
(A, B) Preparation and collection of ribbons of cryo-sections. (A) A ribbon of serial sections is pulled with a gold-coated eyelash (golden arrow) controlled with a double micromanipulator in the cryochamber of the microtome (not shown). Scale bar 0.1 mm. (B) Collection of the ribbon on its support grid after application of the electrostatic press to the section ribbon, with the grid positioned in close proximity to the knife. This step is hereafter referred to as the first charging step. Scale bar 1 mm. (C, C′) Sketch of ribbon collection for optimal adhesion. A first “charge” electrical field is applied after contact with the grid (1). Adhesion is optimized by displacement away from the knife edge (2) and reorientation of the EM grid perpendicular to the antistatic device (3) before applying a second “charge” ionization step (4). (D, D') Adhesion revealed at the macroscopic level: part of the non-adhesive regions of the ribbon, initially white‑silvery (D), turn darker (here purple-grey) upon adhesion after the 2nd step charging (D') that increases the amount of dark surface. The black arrows point to adhesive regions, which are here better visualized where the section lies on a grid bar. (A, B, D) Sections shown here were cut at a feed of 75 nm. Support grids in B: Quantifoil R2/2; in D: Quantifoil R2/1 reinforced with 30 nm carbon. (For interpretation of the references to color in this figure legend, the reader is referred to the web version of this article.)

### 2.3 Support grids

We tested different support films: Carbon Quantifoil R2/1, R2/2, R3.5/1 and S7/2, UltrAuFoil R2/2, on copper or gold (UltrAuFoil) 200 mesh grids (Electron Microscopy Sciences). Quantifoil S7/2 and R3.5/1 were covered with an ultrathin ~2nm(1.7 to 2.8 nm) continuous carbon film, either commercial or evaporated on freshly cleaved mica from a carbon thread (Leica) using an ACE600 evaporator (Leica Microsystems), floated on distilled water, and deposited onto the immersed grids (Passmore and Russo, 2016). Part of the Quantifoil S7/2, Quantifoil R2/1 and UltrAuFoil support films were reinforced with a 30 nm-thick extra layer of carbon evaporated directly on the back side of the grids (sketched in Supplementary Fig. S9). Some UltrAuFoil grids were covered by a ~2nmcarbon layer evaporated on their top surface. These different supports are listed in Fig. 4.

### 2.4 Cryo electron microscopy & tomography

For 2D imaging, grids were mounted in a Gatan 626 cryo-holder and transferred in a JEM-2010F-CRYO (JEOL) electron microscope operated at 200 kV, equipped with a 4k Ultrascan 1000 CCD camera (Gatan). Images were acquired in “search” mode and minimal irradiation (≤ 0.001 e^−^/Å^2^) or in “photo” mode at a pixel size of 0.354 nm/pixel, with an electron dose of ~10–15 e^−^/Å^2^ (1 s acquisition), and a defocus of 2 or 3 μm. For cryo-electron tomography, grids were mounted in Autogrid rings (Thermo Fischer Scientific) and transferred into a Titan Krios G1 or G4 (Thermofischer Scientific), operated at 300 kV and equipped with a Volta phase plate (VPP), a GIF Quantum SE post-column energy filter (Gatan). Images and tilt series were recorded with a K2 Summit (Gatan), K3 (Gatan) or Falcon 4 (Thermofisher) direct electron detector. Atlases were recorded at low magnification (x135, 129.7 nm/pixel) with either ~ 0.1 to 0.15 or 2 to 5 mm defocus. Some atlases were also recorded in a Glacios electron microscope (Thermofisher) operated at 200 kV, with a Falcon 4 camera. Ultra-low dose low magnification tilt series were recorded at ×1400 magnification (pixel size 11.33 nm) with a total dose of 2 e^−^/Å^2^, using Serial EM software (Mastronarde and Held, 2017). Final tilt series recording was performed at a nominal magnification of ×64,000 (2.12 Å/pixel), following the dose-symmetric recording scheme (Hagen et al., 2017) starting from 0°, with an angular range between −60° and +60° and a 2° increment. They were collected with VPP in close to focus conditions (0.25 μm defocus), with an electron dose set to 2.5 e^−^/Å^2^ /image.

### 2.5 Analysis of optical patterns related to section-support film adhesion

Atlas images of the sections were opened in ImageJ. Regions of 24 × 24 pixels, each centered on a single grid hole, were extracted for analysis. To smoothen, the images were upscaled fourfold using bicubic interpolation. Radially averaged normalized intensity profiles were then calculated for each hole using the Radial Profile plugin for ImageJ (https://imagej.net/ij/plugins/radial-profile.html). For averaging, galleries of the 24 × 24 pixel hole-centered regions were collected for each type of pattern and assembled into image stacks. Stacks were aligned with the Template Matching plugin (https://sites.imagej.net/Template_Matching/;https://www.pnas.org/doi/10.1073/pnas.1106377109) using a 16 × 16 pixel central region as the alignment template, the normalized cross-correlation coefficient with subpixel registration, and bicubic interpolation. After alignment, the average intensity Z projections of stacks were calculated and radial intensity profiles were obtained using the same procedure as for individual images. The radial intensity profiles were plotted using GraphPad Prism version 11.0.2.

### 2.6 Image denoising. Tomogram reconstruction and denoising

Some of the 2D images (Fig. 5A) were denoised using wavelet filtration in ImageJ (“A trous” plugin with k_1_ = 20, k_n>1_ = 0). Tomograms were reconstructed with the etomo program of IMOD (Kremer et al., 1996). For reconstructions of low magnification-low dose tomograms, a SIRT-like filter, equivalent to 10 iterations, was applied. Denoising of high magnification tomograms (Fig. 5B) was performed with the deep learning network available in the Warp (Tegunov and Cramer, 2019) software package. For training data, dose-fractionation frames of the tilt series were aligned using the IMOD Alignframes tool, resulting in three tilt series: the complete one consisting of tilt images containing all frames for each tilt angle (*complete*); a tilt series containing even frames for each tilt angle (*even*); and a tilt series containing odd frames for each tilt angle (*odd*). Tilt series alignment was performed on the *complete* series using the patch tracking of etomo, and used to align the *odd* and *even* series. Tomograms were reconstructed in etomo using weighted back projection with the standard ramp filter. The Noise2Map network of Warp was trained on the *even* and *odd* reconstructions, starting with the initial model noisenet3dmodel_256 using the following parameters: dont_augment, dont_flatten_spectrum, learningrate_start 1E-05, iterations 40,000, as described in (Fatmaoui et al., 2026).

## 3 Results

### 3.1 A two-step charging procedure for improved section attachment

We recall in Fig. 1A, B the experimental procedure to generate and collect a ribbon of cryo-section using a double micromanipulator, which lets one control the generation and collection of millimeter-long ribbons (Studer et al., 2014). For attaching the ribbon to its support grid, electrostatic charging, with the use of an electrode (Leica EM Crion) is now preferred to mechanical pressing, as it provides highest efficiency without risk of damaging the sample (Pierson et al., 2010). To optimize the Crion charging efficiency and attachment of the sections, we introduced a two-step charging procedure sketched in Fig. 1C, C'. It begins with a first electrical field pulse applied by the electrode, while the grid is positioned close to the knife under the section ribbon (Fig. 1C), as it has been routinely performed (Pierson et al., 2010). Observing the grid in the microtome binoculars, we noticed that the first pulse leads to the appearance of small darker purple‑gold or purple-grey areas, indicative of locally adhering regions: ultrathin sections typically present a silver reflection color (expected for 50–80 nm-thick sections). Close contact between sections and their support films results in a change of light reflection: non or poorly adhesive regions remain silver (Fig. 1D), whereas adhesive ones become shiny and purple‑gold/purple-grey (Fig. 1D’, Supplementary movie, Supplementary Fig. S2). This phenomenon is due to the change of thickness of the reflecting object: adhering sections come into contact with their carbon support, leading to a single thicker reflecting object, which shifts the reflection color to purple‑gold/purple-grey. We refer to this reflection color change which occurs during contact formation as “melting”. After the first charging step, the grid is pulled away from the knife edge and rotated so that it lies perpendicular to the electrode; a second electrical field pulse is finally applied before the grid is transferred out (Fig. 1C'). Under this second charging step, the small adherent purple‑gold/purple-grey areas initially visible expand: the ribbon is flattened against its support film, previously poorly adherent regions shift to achieve better contact with the support, and the “melting” process propagates. Depending on electrostatic conditions, the additional charging pulse might need to be maintained for several seconds, to maximize the contact areas. Repeating the first pulse may improve adhesion, but only marginally, in contrast to the second-step pulse applied far from the knife and optimally orthogonally oriented. A gallery of ribbons observed after the second step charging is shown in Supplementary Fig. S2. Note that the complete attachment is not possible when the sections are not perfectly flat initially, because of geometric constraints: once an area is attached, it becomes unable to move and may prevent flattening and attachment of neighboring regions. Note that ribbons typically longer than 1.5 mm tend to twist or bend (Supplementary Fig. S1), so that it may be more efficient to collect ribbons in this range rather than long 3 mm ones. It is also important to recall that the quality of the sections is dominated by the knife-sample interaction, which depends on knife and electrode quality. Ideally, sections should be flat, gliding smoothly off the knife. In many cases however, pleats may form. As described by Al-Amoudi et al. (2025, Supplementary Fig. S1), we may distinguish between two types of deformations: (i) large-scale folds, and (ii) local pleats (or wrinkles), referring to small-scale, sharp, and localized distortions. Large-scale folds can, to some extent, be compensated by mechanical manipulation, upon gentle pulling during handling. In contrast, local pleats cannot be fully compensated without risking damage or rupture. A gallery of cryo-EM imaging at low magnification of ribbons attached with either the classical single step charging or this two-step procedure is shown in Supplementary Fig. S3 and S4. This reveals the wide variability in sample quality in both situations. In the worst cases, loss of non-adhering sections or section fragments under liquid nitrogen flow can be observed (Supplementary Figure S3A_1_). Part of the ribbons present numerous pleats and folds, others are flat-looking over long distances (Supplementary Figure S3B_1_), or present alternations of flat and pleated/folded regions (Supplementary Figure S3B_4_). An originally flat ribbon will present few or no pleats, whereas an originally highly pleated ribbon will present many, whether the second-step charging is used or not. But on average, ribbons of sections of similar quality present less pleats when the second-step charging is used. After collection on grid, charging presses and flattens sections against the support film. This is optimized upon the second-step charging, leading to extended adhesive and flat regions.

During the charging we observe local movements of sections on the carbon film prior to definitive adhesion, resulting in the expansion of fully adherent regions that appear as areas of altered contrast (See Supplementary movie). However, the presence of folds and pleats prevents long-range flattening and propagation of adhesion, leading to the formation of non-adhering bubbles trapped between adherent areas. Complete resolution of pleats is therefore impossible, as the growth of fully adherent regions imposes geometric constraints that redistribute the remaining pleats. Whereas pleats generated during sectioning are initially oriented perpendicular to the cutting direction, these “trapped” pleats can also align parallel to the cutting direction (see for example Supplementary Figure S4B_5_). Note that an optimal electrostatic press is required to obtain optimal results: the electrode should be checked beforehand and cleaned regularly using a fiber glass brush tool (Radiospare) to remove oxidized layer from the metal tip. The decrease of air ionization efficiency (with the aging of the electrode for example) can lead to weaker adhesion, which is revealed at the macroscopic level by little change of light reflection upon use of the electrostatic press (Fig. 1D).

### 3.2 Assessment of local (microscopic) adhesion for high throughput tilt series acquisition

Ideally, collected ribbons should not only be flat but also fully adhere to their support; in practice, as mentioned above, sections may present pleats and/or adhere only sparsely to their support, and, in worst cases, not at all. A standard 2D projection image recorded with a typical electron dose of 10–15 e^−^/Å^2^ on a non-adhesive region generally suffers from drift and improper defocusing. A tilt series recorded on a non-adhesive region usually moves independently of the underlying support tracked by the automated tilt series acquisition procedure, and a tomogram cannot be properly reconstructed. It is therefore essential, for optimal throughput, to be able to target efficiently adhesive regions.

At the macroscopic level, samples which do not show long-range silver to purple/gold transition shown in Fig. 1D' and Supplementary Fig. S2 will have too few or no adhesive regions, and must be discarded. Sections obtained with latest-generation diamond knives using a clean, free of ice crystal contamination antistatic device often glide smoothly on the knife's surface, leading to globally flat surfaces at the microscopic level. Pleats that may remain in the final sample are readily identified upon low magnification observation and can be disregarded (see Supplementary Fig. S4, arrowheads). But the observation of a flat region with absence of pleats/folds does not mean that the section adheres to its support. As an example, such a typical low magnification view of a cryo-section is shown in Fig. 2A. To sort out adhesive and non-adhesive regions, we implemented the following two approaches, which let us verify the attachment of areas before setting up tomographic acquisition:

(i) Low magnification (typically ╳1400) recording of a tilt series with ultra-low dose irradiation of the specimen lets us reconstruct the 3D shape of the section and determine its adhesion profile on its support film (Fig. 2B). Adhesive regions of the section appear in close contact with the supporting film (Fig. 2B, green), while non adhesive ones are floating above (Fig. 2B, red). Adhesive and non-adhesive regions can thus be sorted out and high magnification acquisitions can be targeted to adhesive regions (green circles in Fig. 2B). This procedure provides direct validation of area attachment to the supporting film; however, it is time consuming as a low magnification tomogram must be recorded and reconstructed for each region of the sample.
(ii) Atlas recording shows a contrast inversion between adhesive and non-adhesive regions (Fig. 2C, C', Fig. 3), which could correspond to a difference in phase shift. Fig. 2C-C' shows the differential contrast observed on an atlas acquired with a classical defocus, typically in the range of 0.1–0.15 mm, in the region used for low magnification tomography shown in Fig. 2B. The adhesion map of Fig. 2C' is superimposed on the map determined by tomography in Fig. 2A' (white dotted lines), revealing a perfect match. The use of ultra high defocus, typically between 2 and 5 mm, enhances the phenomenon (Fig. 3B). Contrast inversion is observed between adhesive regions and non-adhesive ones or bare support film holes, and appears as a counter-phase feature in radial profiles recorded through holes (or averages of holes), as clearly evidenced in Fig. 3B" (green versus red curves). We tested the reliability of this phenomenon by acquiring high magnification tilt series: among 206 tilt series acquired using this method (i.e. the simple examination of atlases), 175 turned out suitable for high quality tomographic reconstruction (mean residual in etomo <1.0 after automated patch tracking) and further analyses, as illustrated in Supplementary Fig. S6. Adhesive and non-adhesive regions can thus be told apart at a glance. Note that the differential contrast varies not only with the level of applied defocus (Fig. 3), but also with the section and supporting film thicknesses. A gallery of atlases, including of high defocus ones is shown in Supplementary Fig. S7B to illustrate this variability. To investigate the reproducibility of the phenomenon, we recorded atlases of 50 and 75 nm feed ribbons deposited on different types of grids (supports 1, 2, 5, 6, 7 & 8 in Fig. 4), using two different Titan Krios G1 and one Titan Krios G4, all operated at 300 kV. A differential contrast was observed in all atlases. The phenomenon was also observed at 200 kV in a Glacios with parallel beam illumination (illustrated in Supplementary Figure S7A3’). Note that we did not observe this effect using a 200 kV microscope with non-parallel beam illumination (JEOL 2010F, see Supplementary Fig. S3). A wider exploration of the phenomenon, testing different types of microscopes and different imaging conditions would be needed for its full understanding.

**Fig. 3:**
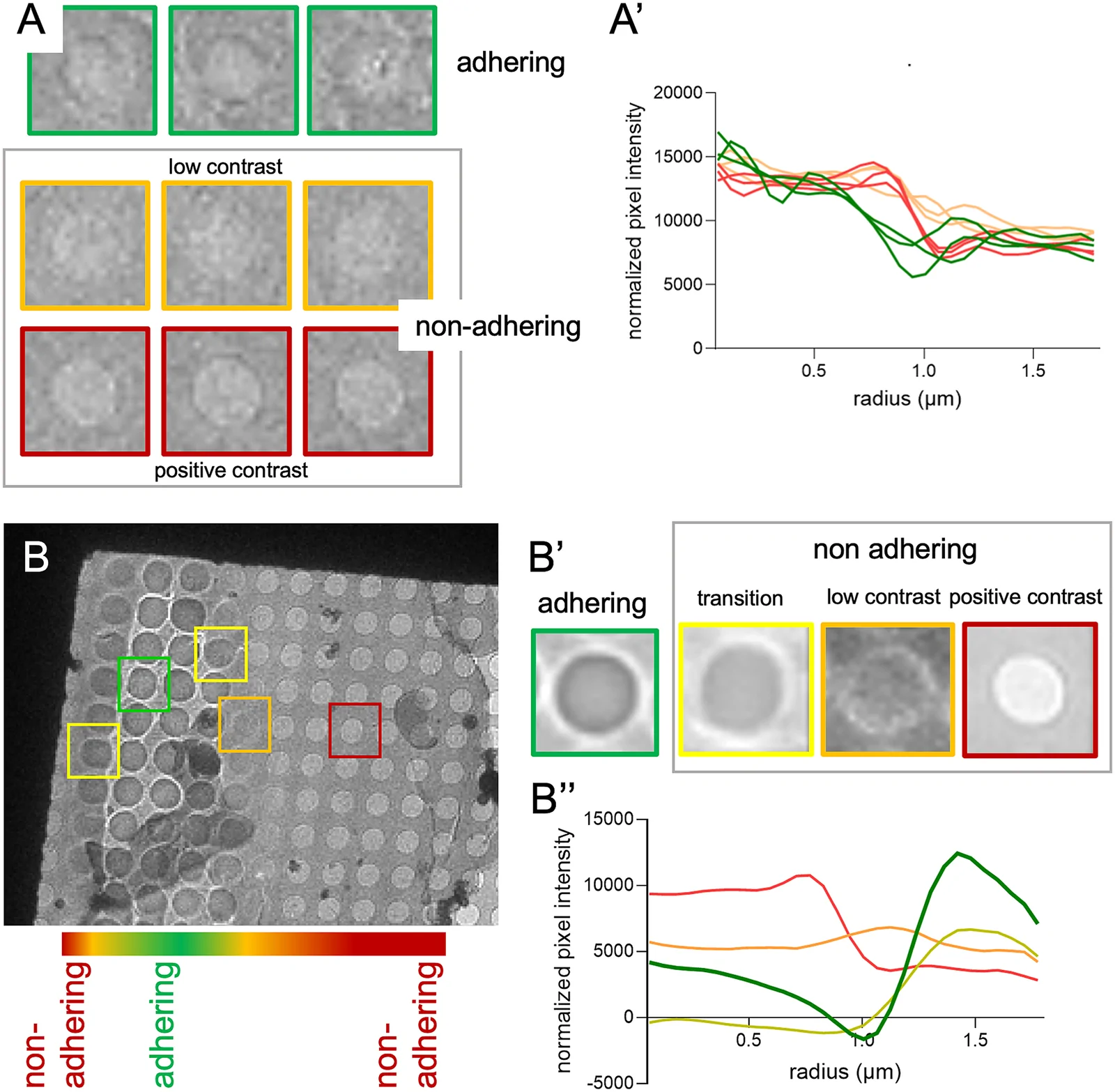
Formation of optical patterns associated with section adhesion to the supporting carbon film. (A) Three types of hole-containing sub-areas representing the different specific optical patterns are selected from the atlas shown in Fig. 2C (defocus ~0.15 mm) and enlarged: adherent areas in green, non-adherent areas in red to yellow. (A') Normalized radial intensity profiles were calculated from three sub-areas representative of each type of pattern. Holes within adherent areas show a contrast rim inside their borders (A', *adhesive*). Non-adhesive regions show two types of patterns: a positive contrast pattern, similar to that found for holes imaged outside the cryo-section, and a low-contrast pattern. We speculate that the low-contrast pattern corresponds to regions where the distance between the section and the supporting carbon film decreases near adherent areas. (B) When the defocus is increased to 2–5 mm, the contrast inversion between adherent and non-adherent areas is enhanced. In addition, a transitional pattern then appears at the border of adherent areas. (B′) Aligned averages of representative images collected from three different atlases are shown for each type of pattern: adhesive (43 areas), transitional (47 areas), low-contrast (26 areas), and positive-contrast (31 areas). Differences between patterns are highlighted by recording radial intensity profiles (B″). Note that this differential contrast results in different hole radii, with a magnifying glass effect (larger holes) in adhesive regions. Experimental conditions: (A) Support grid Quantifoil R2/2; section thickness 75 nm (nominal feed); 1-step charging. (B) Support grid Quantifoil R2/1 reinforced with 30 nm carbon; section thickness 75 nm (nominal feed); 2-step charging. The defocus is ~0.15 mm for A, A'; and ~ 5 mm nominal defocus for B, B′ and B". (For interpretation of the references to color in this figure legend, the reader is referred to the web version of this article.)

**Fig. 4:**
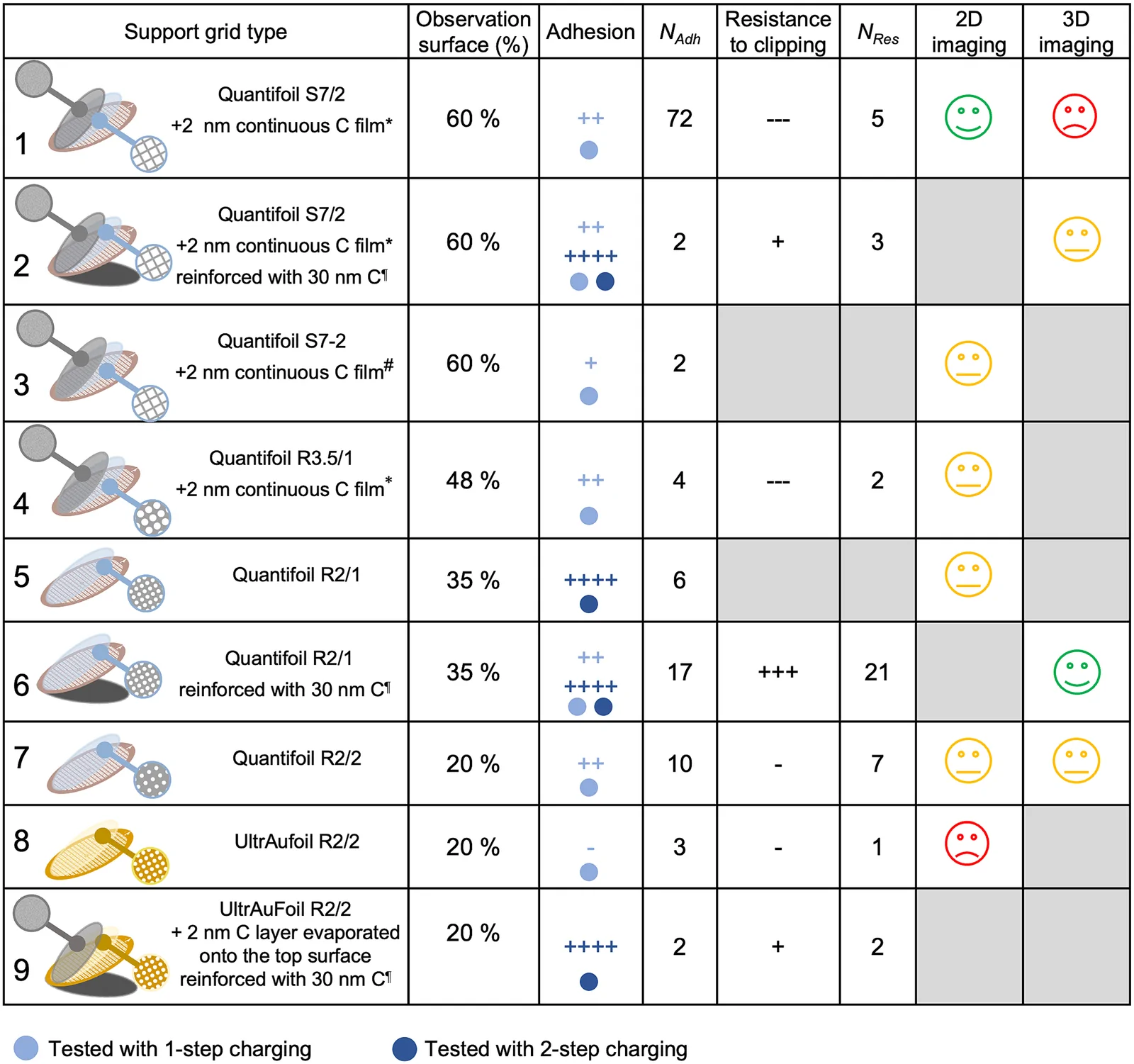
Characterization of different support grids for improved cryo-EM imaging in 2D or 3D (tomography). *, # Commercial holey lattice covered by a continuous ultrathin (~2nm) carbon film evaporated in our Leica ACE (*) or from manufacturer (Quantifoil, #). ¶ Presence of an additional carbon layer evaporated onto the bottom side of the support.  indicates the best performing supports,  the worst ones to be avoided, and  support films that can be used but are not optimal (slightly less adhesive, and/or lower available surface for imaging). The “Observation surface” refers to the amount of hole in the film lattice, disregarding grid bars. The “Adhesion” column integrates all qualitative estimations (macroscopic assessments, beam focusing - see Supplementary Fig. S8 - or random 2D image acquisition), in two cases supported by quantitative surface measurements (see Table 1). « - » means poor adhesion, typically estimated as less than 10% adhesive surface. From «+» to «++++» means data collection varies from possible with low yield (adhesive surface estimated as in the range of 10%), to easy and reproducible (adhesive surface estimated as in the range of 50–70% on average). The « Resistance to clipping » column reports a qualitative estimation of the resistance of the support film after clipping. « - - - / - » means that most/a non-negligible amount of grid square films were broken by the clipping process; « + » means that many grid square films are broken but data acquisition is still statistically feasible; « +++ » means that nearly no grid square films are broken, leading to optimal data acquisition. *N* indicates the number of observed samples in cryo-EM for each type of support grid and for each type of test (Adhesion and Resistance to clipping).

**Fig. 2:**
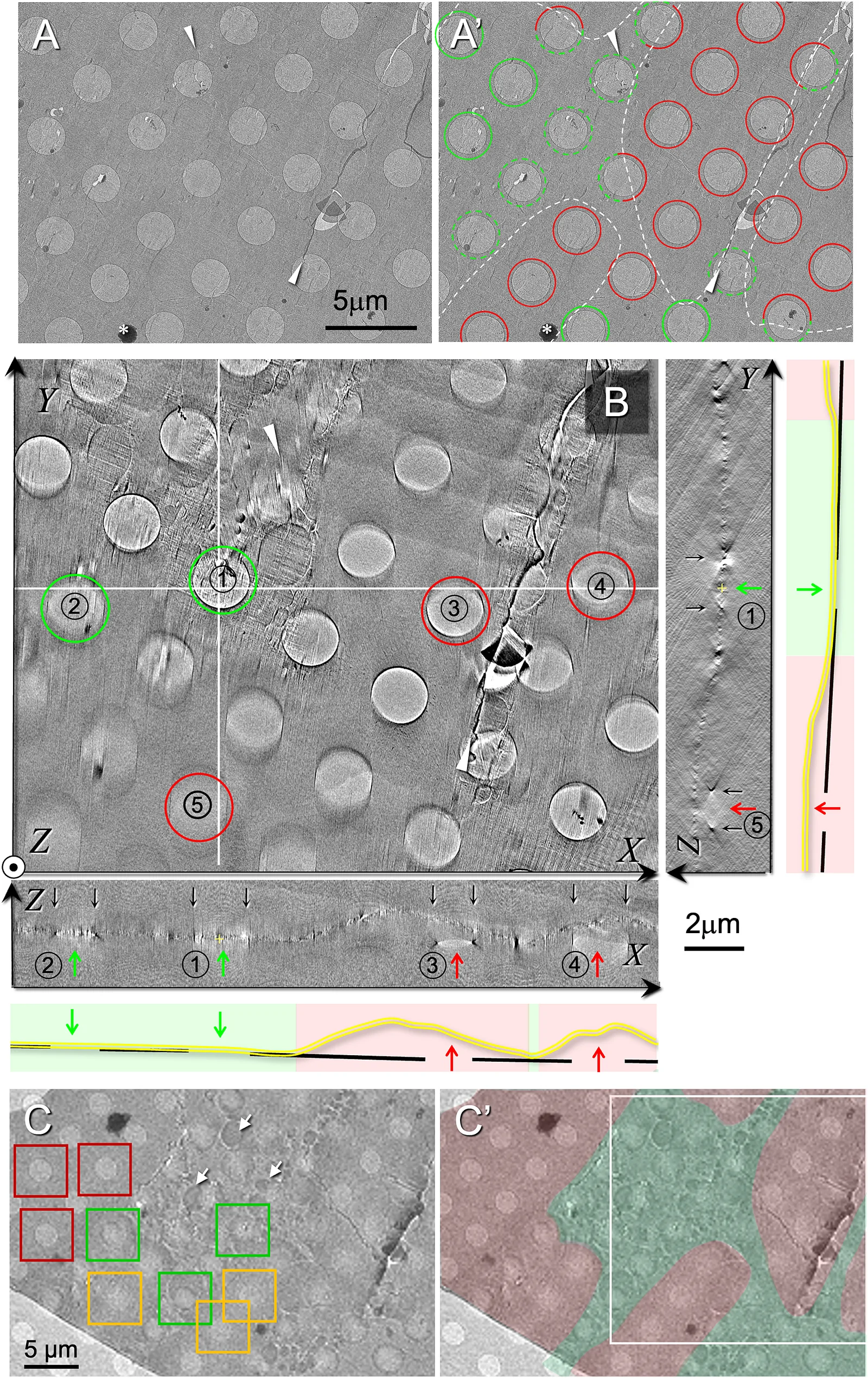
Adhesion tests. (A, A') Low magnification imaging of a section. White arrowheads point to local breaks in the section. White asterisks indicate ice contamination deposited on the section. (A') Adhesion mapping of the area shown by the color code of circles as determined by the tomographic reconstruction shown in (B). Holes circled in green correspond to adhesive areas that can be used for high magnification tomographic acquisitions; a dotted circle indicates that, despite being located within an adhesive region, the area is not optimal (presence of breaks or partially adhesive border). Adhesive/non adhesive borders determined in (C, C′) are shown by the white dotted lines. (B) Low magnification cryo-tomographic reconstruction of the region shown in (A, A'). Virtual slices in XY, XZ and YZ are shown. Holes (1) and (2) (circled in green) are located in adhesive regions in tight contact with the carbon film. The section is floating above holes (3), (4) and (5) (circled in red) located within a non-adhesive area. In XZ and YZ, hole boundaries are pointed by the black arrows. The XZ and YZ profile of the cryo-section on the holey carbon film is sketched. Note that “blurry” regions in the XY slice (e.g. region 2) do not correspond to drift but out of plane areas due to the fact that carbon film is slightly oblique to the XY. A green/red overlay indicates adhesive/non adhesive regions. A series of other virtual slices of the tomogram are shown in supplementary Fig. S5 to illustrate the phenomenon. (C-C') Atlas of the cryo-section (defocus ~0.15mm). The images shown in (A, B) correspond to the white rectangle. A differential contrast is observed in adhesive (coloured in green in C') relative to non-adhesive (in red) regions. Notice that only adherent regions show a high contrast (yolk granules and other structural features present in sections, white arrows). Contrast variations in regions boxed in green/orange/red are analysed in Fig. 3. Support grid: Quantifoil R2/2. Section nominal feed 75 nm. 1-step Crion charging. (For interpretation of the references to color in this figure legend, the reader is referred to the web version of this article.)

At the microscopic local level, perfect adhesion of a section to its support film can also be revealed upon focusing the electron beam on the sample: adhesive regions remain in place, but present bubbling (Supplementary Fig. S8). In contrast, the focused beam creates a hole that propagates rapidly in non-adhesive regions, as it does on unsupported vitreous films. As this test is destructive, it cannot be used directly on ROI when a continuous ultrathin support is used. It is also very local and does not provide a complete mapping of adhesive/non adhesive regions. However, this test can be used for rapid checking of adhesive properties of different support materials (see next section).

### 3.3 Test of different support films for optimal adhesion and resistance to clipping

Different section supports have been used so far to collect cryo-sections, from thin continuous carbon films (Dubochet and Sartori Blanc, 2001), to carbon-covered formvar films (Hsieh et al., 2002) and lattices of holes in carbon films (Bharat et al., 2018) (Eltsov et al., 2018). They must fulfill several requirements which may sometimes be antagonistic: resistance to clipping, no or minimal added noise on recording areas, adhesivity, maximization of surface available for imaging. To our knowledge, there has not been a systematic investigation of any of these properties.

We considered different grid types, listed in Fig. 4. We focused on carbon and gold films with different types of holey lattices:

• Quantifoil round hole carbon arrays R2/1, R2/2, and R3.5/1
• Quantifoil square holes carbon arrays S7/2
• UltrAuFoil round hole gold arrays R2/2

For each type of hole lattice, the amount of available surface Sfor further data acquisition can be calculated from the geometrical parameter of the unit cell of the 2D lattice. For round hole lattices Rx/y, with *x* the hole diameter and *y* the distance between holes (in μm), $S=\frac{\pi {\left(\frac{x}{2}\right)}^{2}}{{\left(x+y\right)}^{2}}$, i.e. 48%, 35% and 20% for R3.5/1, R2/1 and R2/2 respectively. For square lattice, Sx/y, with *x* the hole size and *y* the distance between holes, $S=\frac{x^{2}}{{\left(x+y\right)}^{2}}$, i.e. 60% for S7/2. These values do not take into account the fact that additional parts of the section surface cannot be accessed due to the grid bars, which varies with the mesh size. We used here 200 mesh grids. But 300 mesh ones can also be used and are likely to be more resistant to clipping. Yet, they are not optimal if one needs to access the largest possible sample surface. Note that for 3D tomographic acquisitions, the accessible surface is further reduced, more or less according to the orientation of the grid bars relative to that of the tilt axis.

Too large areas of free-standing sections lead to increased instability under the beam. Large-size hole arrays (S7/2 and R3.5/1) were therefore covered with a continuous ultrathin ~2 nm carbon film, either homemade, evaporated from a carbon thread in our Leica ACE evaporator (Fig. 4^⁎^), or commercially available (Quantifoil S7/2, Fig. 4^#^). We searched to optimize resistance to clipping by testing two different film thicknesses: commercial grids were thus used either directly - with their original 20 nm-thick film – or after evaporation of an extra 30 nm strengthening carbon layer from the copper side of the grid, leading to a total thickness of 50 nm (Fig. 4, Supplementary Fig. S9).

For adhesion tests, we observed (either 2D or 3D imaging) from 2 (UltrAuFoil covered with carbon, Quantifoil S7/2 with continuous commercial film, reinforced S7/2 with continuous homemade film) to 72 (S7/2 with continuous carbon film) sample grids (Fig. 4, *N*_*Adh*_). In the case of UltrAufoil, a few more grids were tried, but we failed to recover sections on account of the lack of adhesion. For resistance to clipping tests, we tested from 1 (UltrAufoil) to 21 grids (Reinforced R2/1) (Fig. 4 *N*_*Res*_). This large discrepancy is due to the fact that we mostly tested the reproducibility of best candidate supports.

Adhesion tests described above (Fig. 1, Fig. 2, Supplementary Figs. S6–8) were performed on ribbons deposited on the different types of grids. The results are synthetized in Fig. 4, which qualitatively integrates all tests, at the macroscopic and microscopic levels. Note that the observed macroscopic color depends on many parameters: hole lattice, nature and thickness of the film, incident light… (see Supplementary Fig. S2). For example, the color change observed with S7/2 lattices is weaker than that observed with R2/1, and R2/2. Systematic microscopic adhesion tests were then performed either by beam focusing on sections regions covering the support film (Supplementary Fig. S8), and/or high defocus atlas mapping (Fig. 3, Supplementary Fig. S7). In the first case, tests were followed by acquisition of 2D images; in the second, atlas recording was followed by acquisition of tilt series. Microscopic adhesion tests show that gold surfaces are the least adhesive ones, with nearly no adhering region found. No 2D image or tilt series could be recorded. The lack of adhesion on gold is confirmed by the fact that sections could be moved on film using an eyelash. Evaporating a thin carbon layer (~2 nm) on UltrAuFoil films recreates an adhesive surface, probably equivalent to that of pure carbon films, although we did not search to quantify it. Note that this was tested only on UltrAufoil grids reinforced by a 30 nm carbon film evaporated on the back side of the grid (Fig. 4, support 9). 2D images acquired randomly on sections deposited on carbon surfaces result in about 60–80% of high-quality images - as characterized by the absence of drift (Fig. 5A). In good agreement, surface measurement on atlases recorded at high defocus show that about 50–80% of a ribbon's surface is adhesive on these films (Table 1). Interestingly, homemade continuous carbon films obtained by carbon thread evaporation at a high-vacuum ($\leq 5.5\;10^{-5}$ mbar) were found more adhesive than commercial ones (Fig. 4, supports 1 and 3 respectively). Note that addition of the continuous homemade carbon layer was tested only with two commercial film grids (S7/2 and R3.5/1); more tests would be needed to confirm this result.

**Fig. 5:**
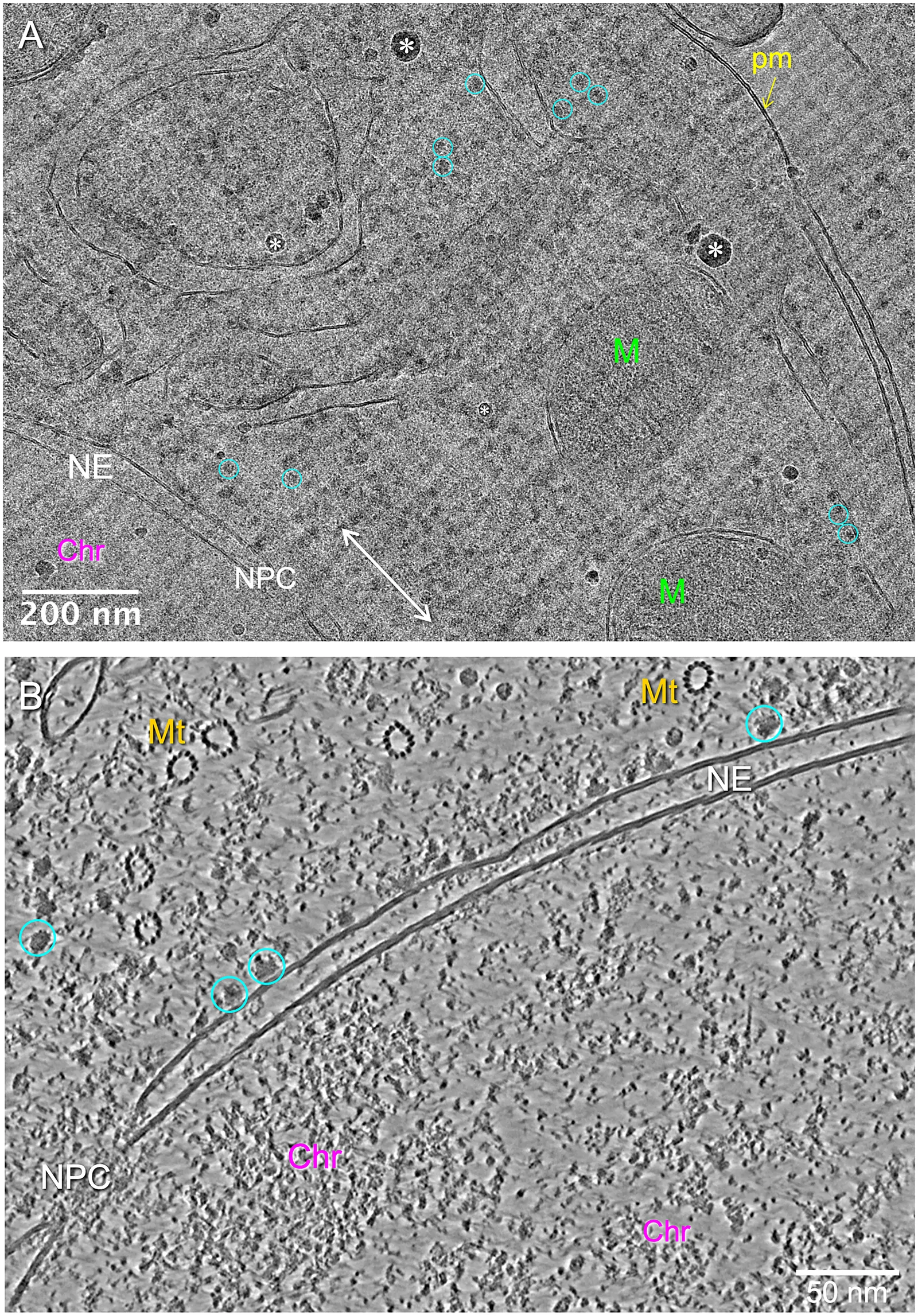
Examples of cryo-EM/ET imaging of the nuclear periphery of Drosophila embryonic brain. NE: nuclear envelope, NPC: nuclear pore complex, M: mitochondria, Mt.: microtubule, Chr: chromatin. A few ribosomes are circled in cyan. (A) Cryo-EM 2D image of a 50 nm-thick section. Some ice contaminations deposited on the section surface are indicated by white asterisks. Cutting direction is indicated by the double white arrow. The image is denoised with ImageJ wavelet filter. (B) Virtual slice (4.25 Å) in a cryo-tomogram of a 50 nm-thick section. The tomogram is denoised using the noise2noise deep-learning network Warp (Tegunov and Cramer, 2019). Experimental conditions: 2 step Crion charging. Support grids: (A) Quantifoil S7/2 covered by 2 nm-thick continuous carbon film, and (B) Quantifoil R2/1 reinforced with 30 nm carbon. (A) Defocus 2 μm. (B) VPP contrast, defocus 0.25 μm. (For interpretation of the references to color in this figure legend, the reader is referred to the web version of this article.)

**Table 1:** Amount of adhesive surface measured from high defocus atlases using either 1 or 2-step electrostatic charging (Crion) on different types of grids. *N* indicates the number of ribbons analysed in each case. Note that the first line compares two types of S7/2 grids: support 1 (S7/2 + continuous homemade carbon film) and support 2 (S7/2 + continuous homemade carbon film, reinforced), used respectively for 1-step and 2-step Crion charging experiments. Although the two types of supports grids are not identical, it is very unlikely that reinforcement of the square lattice at the back side of the grid modifies adhesion on the top side.

	1-step Crion	*N*	2-step Crion	*N*
S7/2 + continuous carbon film (homemade*) (supports 1 & 2 in Fig. 4)	28%	1 (support 1)	69% ± 14	2 (support 2)
R2/1 (support 6)	10.8% ± 4.9	3	65% ± 18	13

Resistance to clipping was tested by simple examination at low magnification (Supplementary Fig. S10). All commercial films showed multiple broken areas, leading to loss of sections in many cases. However, films with smaller holes and/or hole density (i.e. smaller observable surface) are more resistant, with R2/2 arrays more resistant than S7/2. We initially found that strengthening of the films with the addition of an extra 30 nm carbon layer from the back side improves resistance to clipping for Quantifoil S7/2 and UltrAuFoil grids. We then directly applied this procedure to R2/1 grids, which were our preferred choice for tomography, and observed a similar effect: nearly 100% recovery of the grid surface was achieved (Supplementary Fig. S10D). However, it should be noted that, even after this reinforcement, grids with large-hole arrays (S7/2) remain more fragile, with a high probability of breakage.

### 3.4 *Adhesive* versus non-adhesive surface measurements in different experimental conditions

Atlases (Figs. 2C & 3, Supplementary Fig. S7) provide a direct visualization of adhesive versus non-adhesive surfaces, which makes it possible to quantify adhesion. In Table 1, we report the ratio of adhesive/non-adhesive surface areas measured on atlases recorded in some of the experimental conditions explored here, i.e. after one or two step Crion electrostatic charging, and for two types of support films. Despite the limited number of conditions reported in Table 1, these measurements are consistent with our qualitative estimations of the benefit of the additional charging step (color change upon 1-step versus 2-step charging, amount of 2D drift-less image acquisition): in the case of reinforced Quantifoil R2/1 (support 6 in Fig. 4), the second step charging increased the amount of adhesive surface. The measurements performed on S7/2 grids with a continuous carbon film also agree with this observation, although the strength of the comparison is limited by the small number of measurements and a difference in reinforcement (additional 30-nm-thick carbon layer deposited on the back of the grid - see Supplementary Fig. S9 - for the 2-step charging measurements). As no effect of the reinforcement on adhesion was indicated by qualitative estimations (for supports 1 versus 2 as well as 5 versus 6, see Fig. 4), we consider that these measurements provide further support for improved adhesion after 2-step charging. Note that, within a sample, there exists local variability: after 1-step Crion charging, some grid squares may present no adhesive regions (0%), whereas others may reach about 50% of adhesion; likewise, after 2-step Crion charging, we found grid squares with as low as 10–15% and up to nearly 95% of adhesive surfaces. This is illustrated in Fig. 2, showing a relatively well adhering region (42% of adhesive surface) of a sample for which only 1-step Crion charging was used.

Altogether, our tests lead to two recommendations: (i) for 2D imaging in side entry microscopes – for example used for screening of the structures and initial vitrification assay for bulky samples like organs or organisms - we recommend S7/2 carbon lattices covered by an ultrathin continuous carbon film. The S7/2 square lattice provides the highest imaging area and the continuous carbon underlying the sections ensures optimal adhesion everywhere; (ii) for 3D tomographic imaging in high end microscopes requiring clipping of the sample grids, best results in terms of throughput of high-quality tilt series were obtained with R2/1 Quantifoil carbon lattices reinforced by a 30 nm carbon layer. Reinforced R2/2 Quantifoil carbon lattices can be expected to be reliable as well, altogether adhesive and clipping-resistant, but they offer smaller surfaces for data acquisition.

## 4 Discussion

In this work, we establish an optimized workflow for the preparation of cryo-sections suitable for cryo-electron tomography (cryo-ET), with a particular focus on enhancing section adhesion and enabling reliable identification of well-adhered regions. To maximize adhesion, we introduce a second electrostatic charging step during section collection and systematically evaluate different support grids with respect to their adhesion performance and mechanical stability during clipping. In addition, we present a rapid assessment protocol that allows immediate identification of regions within a section ribbon that are optimal for high-quality tilt series acquisition.

The introduction of real-time macroscopic assessment enabled us to experimentally evaluate the standard operating recommendations for the electrostatic press. We observed that charging efficiency is low when the grid is positioned in close proximity to the diamond knife. We hypothesize that the conductive surface of the knife base attenuates the electrostatic field required for effective section adhesion. To address this limitation, we implemented an additional charging step performed in an optimized geometry and at a sufficient distance from bulky conductive components within the cryo-microtome chamber.

The further analysis by low magnification tomographic reconstruction (Fig. 2B) and atlas recording (Figs. 2C & 3, Supplementary Fig. S7) both yield a complete adhesion map of the sections, the latter being altogether exhaustive and fast. They both show that cryo-sections deposited on the support film consist of a mosaic of adhesive and non-adhesive domains. Even for the best support films tested here, there exist non adhesive “bubbles” (Fig. 2, Fig. 3, Supplementary Figs. S5–7), not suited to tomographic acquisitions or even high-quality 2D image recording. We suspect that this is due to (i) the non-perfect flatness of the sections during generation, which would lead to local hills and valleys precluding “melting” (adhesion) propagation; and/or (ii) the presence of adhesive and non-adhesive patches on the carbon film. Carbon films are indeed heterogeneous supports with in particular patches of sp^2^ and sp^3^ hybridizations (Chu and Li, 2006) (Kim et al., 2021). Their relative amount changes with evaporation conditions in association with changes in mechanical and electrical properties of the film (Kim et al., 2021). This could also explain why different results are obtained with different carbon sources (Fig. 4, supports 1 and 3), with homemade continuous carbon films performing better than the commercial continuous carbon from Quantifoil. Conductive gold surfaces proved poorly adhesive.

Whereas the nature of the surface plays a role in adhesivity, the lattice type plays a role in (i) sample stability under the beam, and (ii) resistance to clipping. As mentioned above free-standing sections over large holes are poorly stable under the beam and cannot be used directly. Free standing sections over small (2 μm) holes are also slightly less stable under the beam than sections adhering to a continuous film, leading to less driftless 2D images. This could be due to the presence of nano tears in some regions, which could propagate within free-standing sections and be fixed on adhesive films. We did not test the influence of the presence of a continuous film for tilt series acquisition on R2/1 or R2/2 lattices. But, with a very high yield of acquisition of tilt series suitable for tomographic reconstruction on bare R2/1 lattices, its absence can be expected of little consequence. In addition, it could introduce noise. To resist clipping reproducibly, commercial support films need to be thickened to 50 nm. Importantly, the additional carbon layer must be deposited from the back side of the grid. We hypothesize that this configuration provides increased mechanical resistance by reinforcing the contact between the support film and the grid bars (Supplementary Fig. S9). In addition, the lattice type plays a role: the higher surface/hole ratio of the support film, the more resistant to clipping it is. S7/2 lattices present the highest observation surface (60% of the total surface), but – even thickened to 50 nm - remain fragile. They also often present long-range undulations which complicate tilt series acquisition. However, covered by an ultrathin continuous carbon film, S7/2 lattices, due to their large and adhesive imageable areas, are the best choice for 2D imaging in side-entry microscopes for which no clipping is required. R2/1 Quantifoil grids reinforced with carbon turned out to provide stable tilt series recording on well adhering sections. They also present excellent resistance to clipping (nearly no loss). They should be preferred to reinforced R2/2 (not tested with reinforcement layer) on account of their higher imageable surface (35% versus 20%). Examples of 2D images and a reconstructed cryo-tomogram respectively acquired on these two types of support are shown in Fig. 5A, B. This study focused on the most commonly used support films; however, even within this limited set, a substantial diversity of adhesion properties was revealed. Other films may also be worth exploring, such as SiN membranes with or without graphene coatings (e.g. supports from CryoSilico and DENSsolutions), graphene oxide or formvar.

CEMOVIS is an alternative to cryo-FIB milling especially adapted to tissues and even whole organs or organisms which can be vitrified over large scale, typically up to 200 μm deep (Sartori et al., 1993; Leforestier et al., 2014) in the absence of any cryoprotection by high pressure freezing. Another advantage is the possibility to generate ultrathin samples, well below 100 nm if desired (down to 20–30 nm Ng et al., 2020; Al Amoudi et al., 2025), and serial sections. It can also be used as structure screening tool, with rapid sampling of large areas. Typically, a successful sectioning day results in 4 to 5 grids covered with a ~2mm-long and ~50μm-wide ribbon. Considering that 30% of the surface is lying over grid bars, about 100 to $120\times 10^{3}$ and 170 to $210\times 10^{3}$ ${\mathrm{\mu m}}^{2}$ are thus available for imaging on R2/1 (35% of carbon-free area) and S7/2 (60% of carbon-free area) grids respectively.

Together with stable generations of cryo-ultramicrotomes (Leica UC6/FC6 and UC7/FC7) and diamond knives (Diatome CEMOVIS 35 and 25), a series of progresses have been implemented in the CEMOVIS workflow over the years: (i) the use of double micromanipulators, which, although not fully automating the delicate generation and collection of ribbons, considerably alleviate the procedure (Hsieh et al., 2006)(Studer et al., 2014); (ii) performing the process inside a controlled environment; (iii) the use of an electrostatic press instead of a mechanical one (Pierson et al., 2010). To limit contamination, dry glove boxes have been used (Pierson et al., 2010) but introduce an extra obstacle in the handling of the sections. We opted for a partly dehydrated room, at 20 to 25% relative humidity, which results in an optimal balance between contamination and comfort of the user. Cutting at the highest possible temperature below devitrification (−140 °C) (Ng et al., 2020; Al Amoudi et al., 2004), use of low angle knives (25°) (Han et al., 2008) and minimization of the width of the block face (typically 50–75 μm) (Al Amoudi et al., 2005) optimize section generation and minimize crevasses and/or compression artefacts. Our present work goes a step further, leading to high throughput cryo-ET. In our hands, the amount of tilt series suitable for optimal tomographic reconstruction was not different from that obtained with thin film samples. Typically, we obtain 50 tomograms per day, of which more than 45 are suitable for reconstruction.

This also opens a way toward serial tomography. We did not attempt here to record serial tomograms. In most samples, there are good chances that a given ROI found in an adhesive region of a section is also adhesive in the next section, as already noticed by Ng et al. (2020). However, considering the available imaging surface of R2/1 lattices (35%), chances to access successive sections of a ROI are low. Highest observation surfaces can be obtained using continuous ultrathin support films directly deposited on grid bars: carbon film-coated parallel-bar grids were thus successfully used by Ng et al. (2020) to obtain serial tomograms of *S. cerevisiae*. However, such support films are likely to be too fragile to be clipped for tomographic acquisition in high end cryo-microscopes. S7/2 lattices tested here, even strengthened by additional carbon layer are still not resistant enough and wavy, reducing high-quality tomographic acquisition throughput. We did not test tilt series recording on sections deposited on Quantifoil R3.5/1 lattices (possibly covered with an additional ultrathin continuous film), but this could be an interesting solution, leading to higher probability of serial tomographic acquisitions.

## CRediT authorship contribution statement

**Fatima Taiki:** Writing – review & editing, Writing – original draft, Methodology, Investigation, Conceptualization. **Helmut Gnaegi:** Writing – review & editing, Methodology, Investigation, Funding acquisition, Conceptualization. **Diana Grewe:** Writing – review & editing, Methodology, Investigation, Conceptualization. **Nils Marechal:** Investigation. **Mikhail Eltsov:** Writing – review & editing, Writing – original draft, Supervision, Methodology, Investigation, Funding acquisition, Conceptualization. **Amélie Leforestier:** Writing – review & editing, Writing – original draft, Supervision, Methodology, Investigation, Funding acquisition, Conceptualization.

## Declaration of competing interest

HG is founder and employee of Diatome, a company manufacturing diamond knives for ultramicrotomy. The authors declare that they have no other known competing financial interests or personal relationships that could have appeared to influence the work reported in this paper.

## Data Availability

Data will be made available on request.
