# Supplementary material for: Improving adhesion and targeting of adherent areas for high-throughput cryo electron tomography of vitreous sections
Source: J Struct Biol X. 2026 Oct 20;14:100167. doi: 10.1016/j.yjsbx.2026.100167 (PMC13743409; doi:10.1016/j.yjsbx.2026.100167)
Supplement: Supplementary file 1 [file mmc1.pdf]

Supplementary Information

Fatima Taiki[1], Helmut Gnaegi[2], Diana Grewe[3], Nils Marechal[4], Mikhail Eltsov[4], Amélie Leforestier[1]

[1] Laboratoire de Physique des Solides (LPS), CNRS, Université Paris-Saclay Orsay, France
[2] Diatome Ltd, Helmstrasse 1, Nidau, Switzerland
[3] Buchmann Institute for Molecular Life Sciences, Goethe University, Frankfurt am Main, Germany
[4] Centre for Integrative Biology (CBI), Department of Integrated Structural Biology, IGBMC, CNRS, Inserm, Université de Strasbourg, Illkirch, France

Correspondence
amelie.leforestier@universite-paris-saclay.fr; eltsovm@igbmc.fr

Supplementary Figures S1 to S10

Supplementary Figure S1

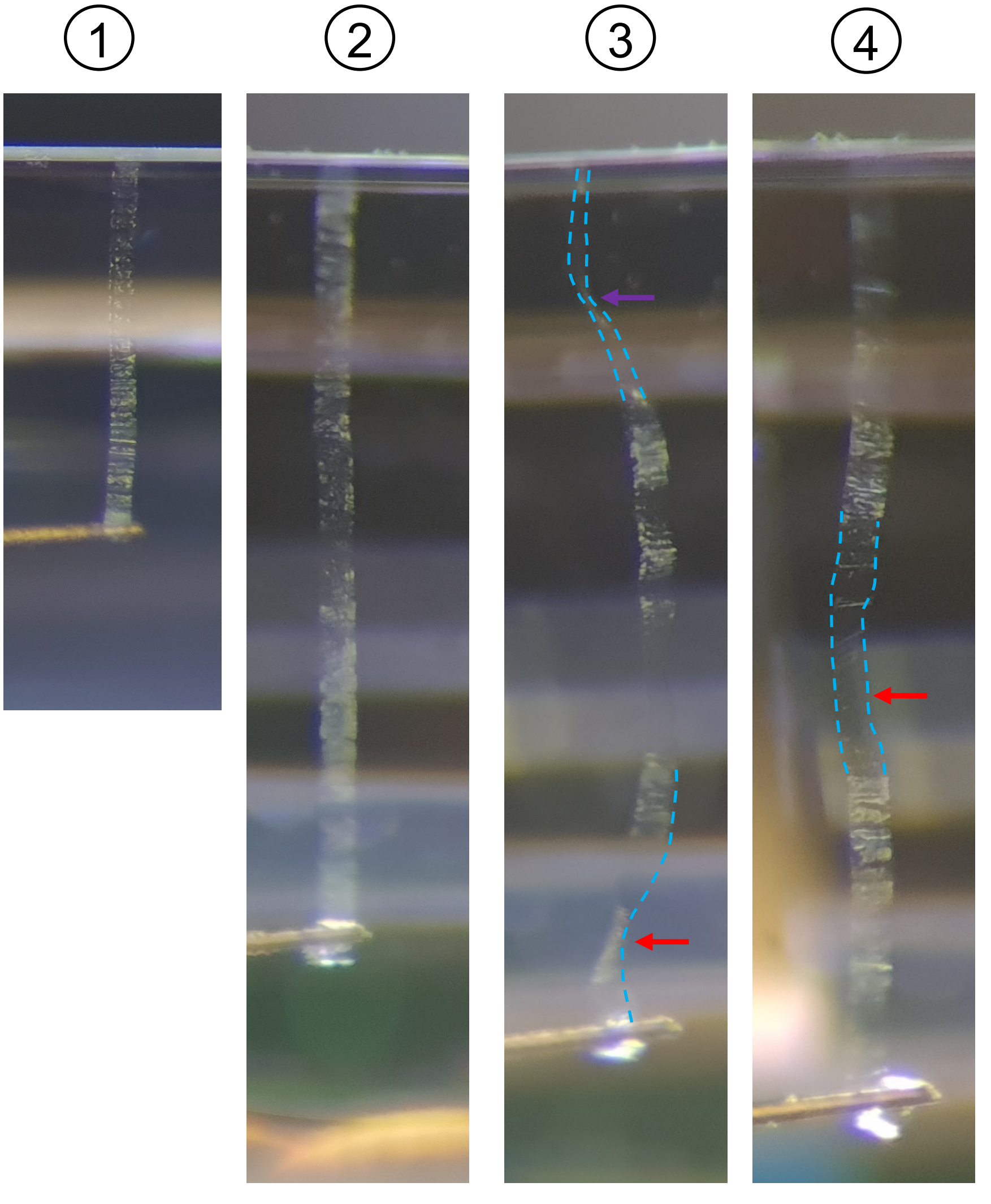

Figure S1. A few examples of ribbons during the generation process. Even and flat short ribbons are easily generated (1, 2). Longer ribbons (3, 4) tend to twist or bend.

Supplementary Figure S2

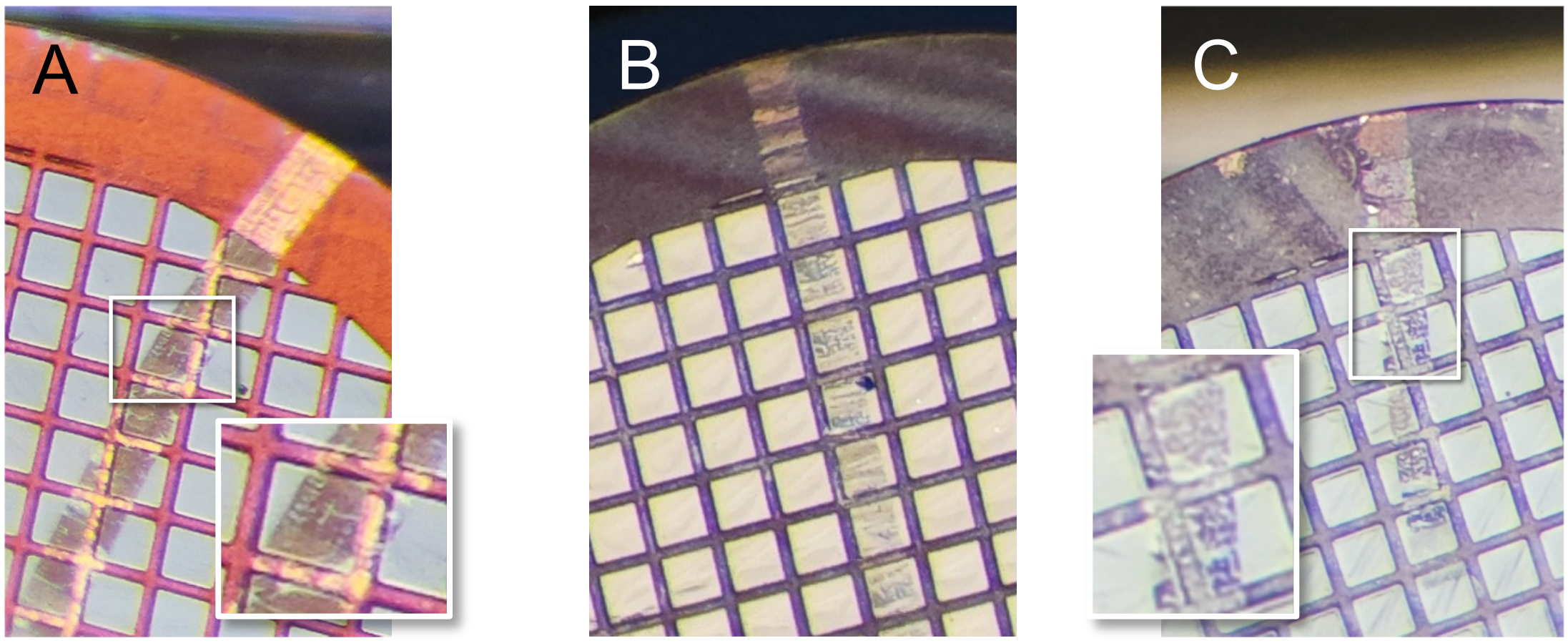

Figure S2. Gallery of ribbons after 2-step charging. Adhering regions appear darker than the white-silvery non-adhesive ones and may present a variety of colours (from purple-gold to purple-grey or brown-grey) according to the grid batch, type of supporting carbon film and grid orientation in the microtome (variable light reflection).

75 nm feed section ribbons are deposited on Quantifoil R2/2 (A, C) and R2/1 (B).

Supplementary Figure S3

Figure S3. Examples of cryo-EM low magnification imaging of ribbons collected with one-step (A) or two steps (B) electrical field charging (Crion electrode used in charging mode) on different support grids. Feed = 75 nm.

In (A1) missing regions resulting from large non-adhesive regions were flown away by liquid nitrogen during transfer (red asterisks). Other ribbons/regions remain on grids (A1 white arrowheads, A2-4, B).

Ribbons shown here are deposited on support films Quantifoil R3.5/1 covered by ultrathin continuous carbon evaporated from carbon threads (A1, A2), R2/2 (B1), R2/1 (B3) and S7/2 covered by ultrathin continuous carbon evaporated from carbon threads (A3, A4, B2, B4). B3 support film was reinforced with 30 nm carbon on the back side of the grid.

A1-4 and B1, 2, 4 were not clipped and observed in JEOL 2010F. B3 was clipped and observed in Titan Krios G1.

A

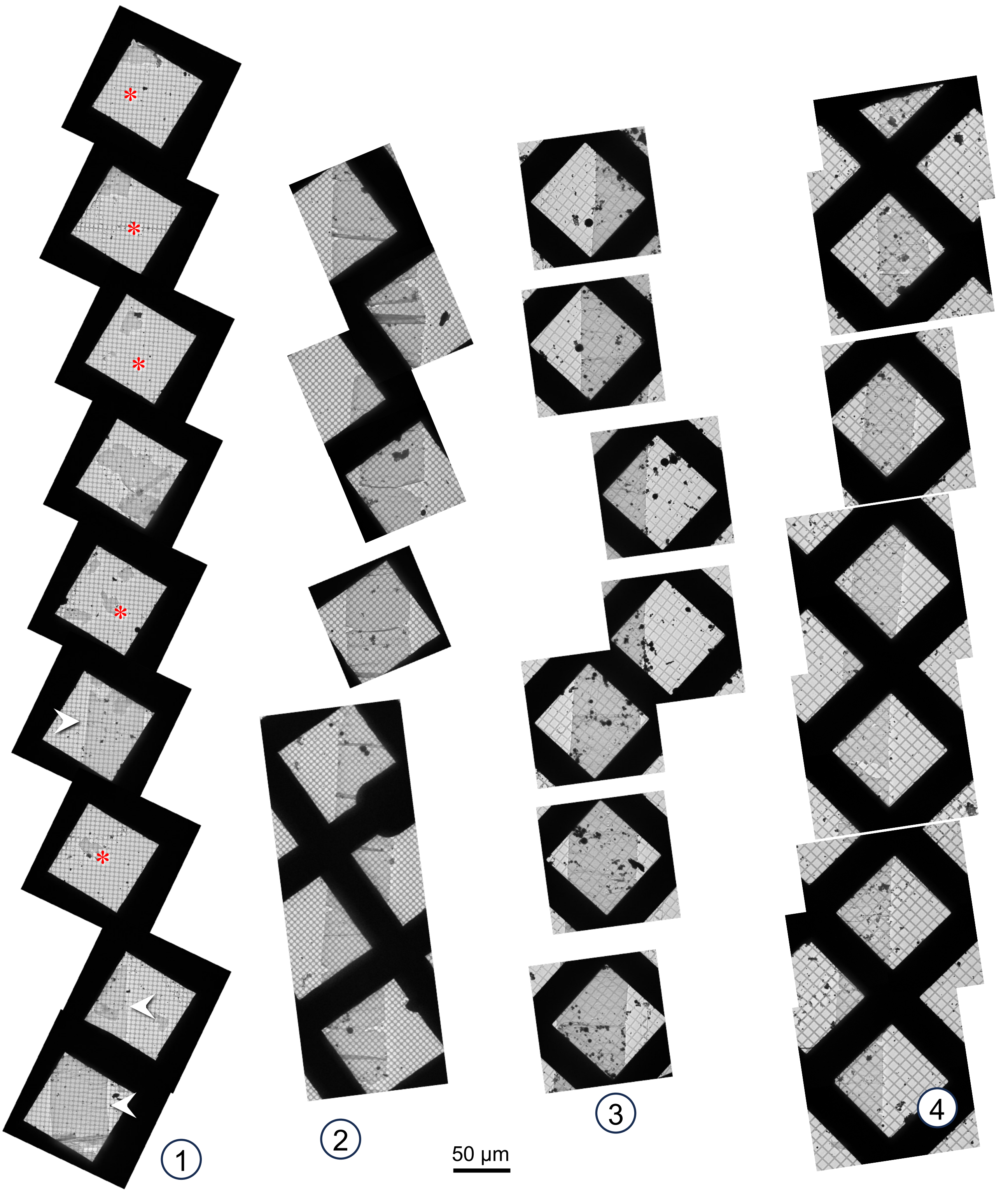

B

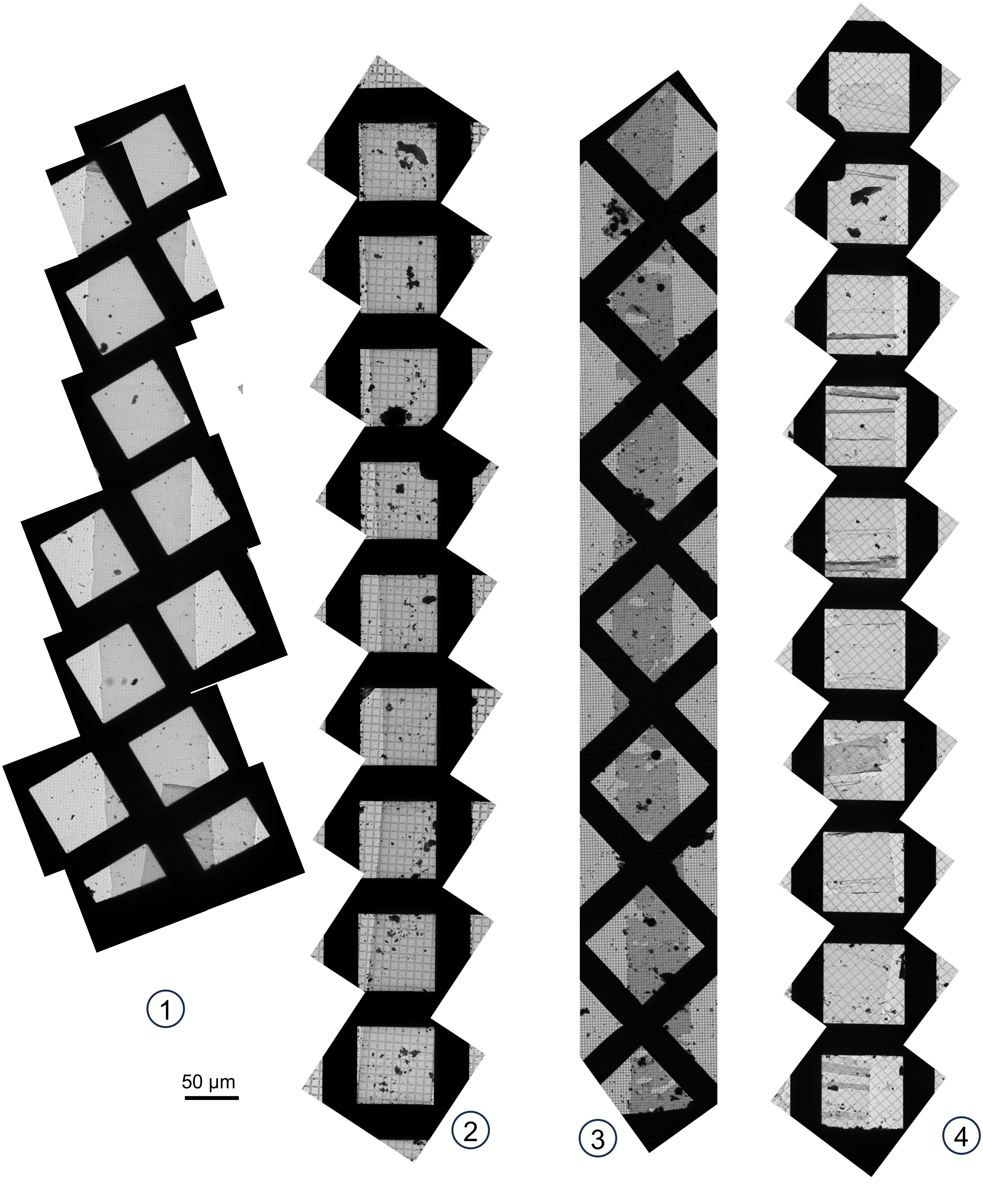

Supplementary Figure S4

Figure S4. (A) Low magnification images of cryo-sections after 1-step electrical field charging (Crion electrode used in charging mode). Feed = 75 nm.
Sections 1-5 are deposited on Quantifoil S7/2 covered with ~2 nm continuous carbon film; section 6 is deposited on Quantifoil R3.5/1.
(B) Low magnification images of cryo-sections after 2-step electrical field charging (Crion electrode used in charging mode). Feed = 75 nm (sections 1-4) and 50 nm (sections 5, 6). Sections (1, 2, 6) are deposited on Quantifoil S7/2 covered with ~2 nm continuous carbon film; sections 3 & 4-5 are deposited on Quantifoil R2/1 & R2/2 respectively.

Yellow arrowheads point to some pleats/folds. Asterisks indicate some ice crystal contaminations deposited on the sample.

A

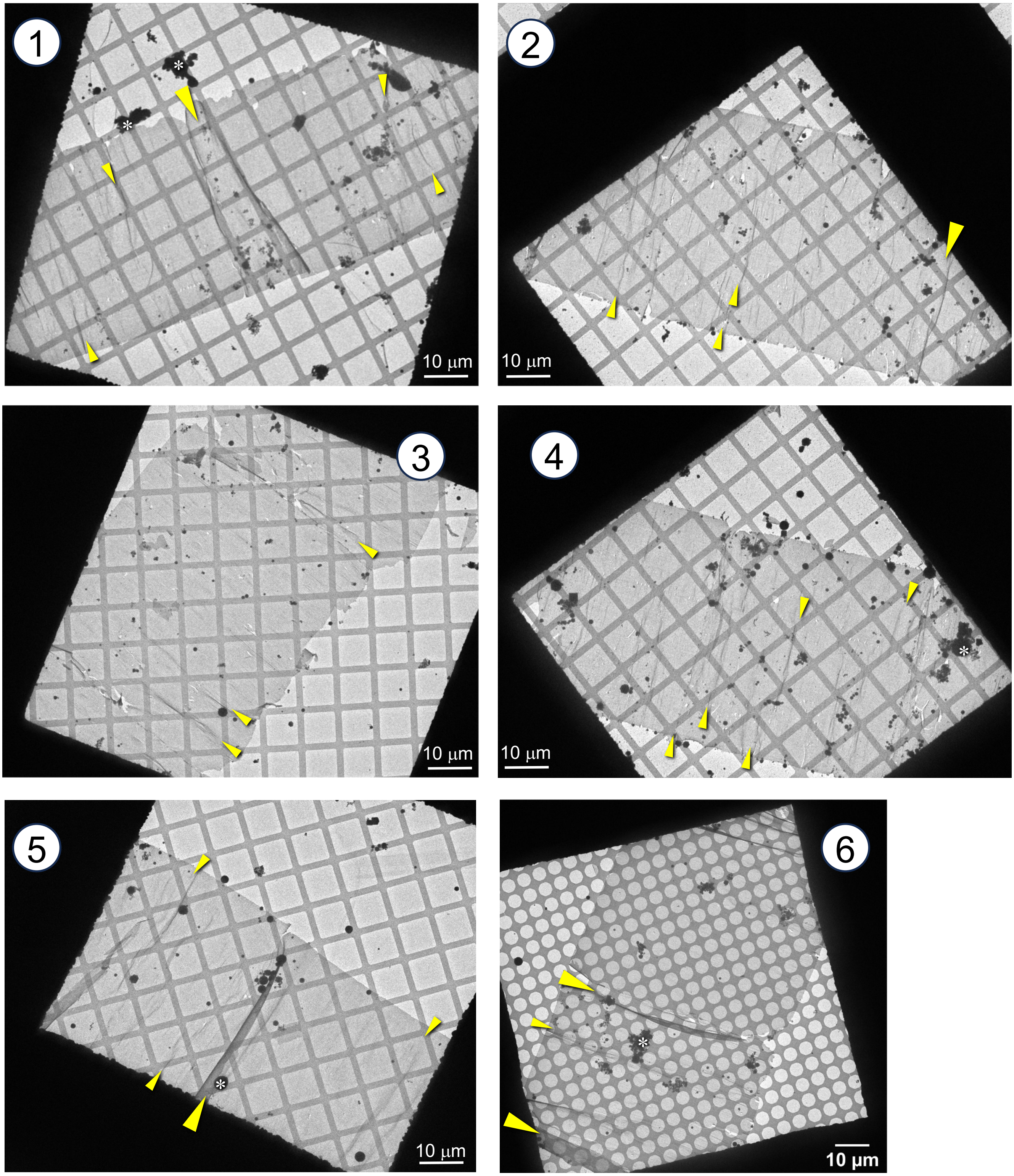

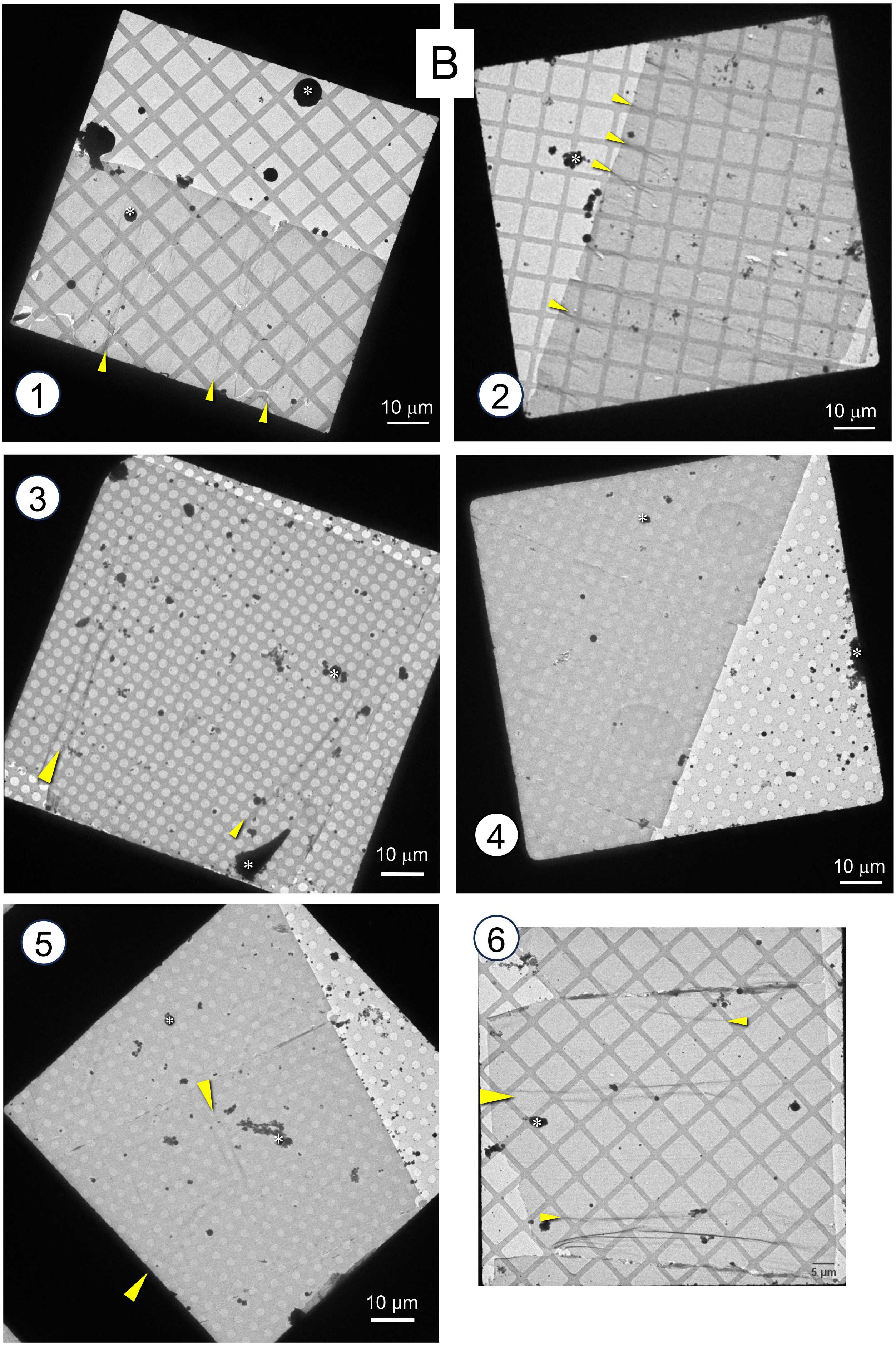
B
1
10 µm
2
10 µm
3
10 µm
4
10 µm
5
10 µm
6
5 µm

Supplementary Figure S5

Figure S5. Low magnification tomography of vitreous sections (75 nm-thick section deposited on Quantifoil R2/2. 1-step charging procedure).
(A) Low magnification 2D imaging of a section. Adhesive regions suitable/unsuitable (green/red circles) for high magnification tomographic acquisitions, determined from the tomographic reconstruction shown in (C), are indicated by the green/red circles. Green dotted circles indicate adhesive regions but suboptimal for high magnification tomographic acquisitions on account of breaks of proximity to non-adhesive regions (1, 6, 7). White arrowheads point to local breaks in the section. The white asterisk indicates ice contamination deposited on the section.
(B) Isosurface rendering of cross section views of the cryosection and its support film, from boxes in white shown in C (Z=114 & 124). Left: adhesive region. Right: transition between an adhesive region (9) and a non-adhesive one (8). The holes of the supporting carbon film are indicated by the double arrows.
(C) Gallery of virtual slices (22.7 nm) in the XY and XZ directions. In each case, the XZ section is shown by the white line drawn on the corresponding XY one. Note that the blurry aspect locally observed in many subregions of the XY planes is not related to drift, but to the fact that the sample is not perfectly oriented parallel to XY, so that the different regions are not imaged in the same *Z* plane.
Adhesion is characterized by close contact between the carbon film plane and the section. The section and the carbon film hole edge are then imaged in the same XY plane (1, 2, 6, 9, left half of 7). In contrast, non adhesive regions are floating above the carbon film and are imaged at different Z heights (3, 4, 8, right side of 7): for example, in region 8, the cryosection is imaged in plane 58, whereas the carbon film with its holes is imaged in plane 124.
The carbon film and the section's cross sections are underlined locally by the dotted line and the yellow double curve respectively.
(A) is reproduced from Figure 2A'; the Z=114 slice in (C) is also shown in Figure 2B.

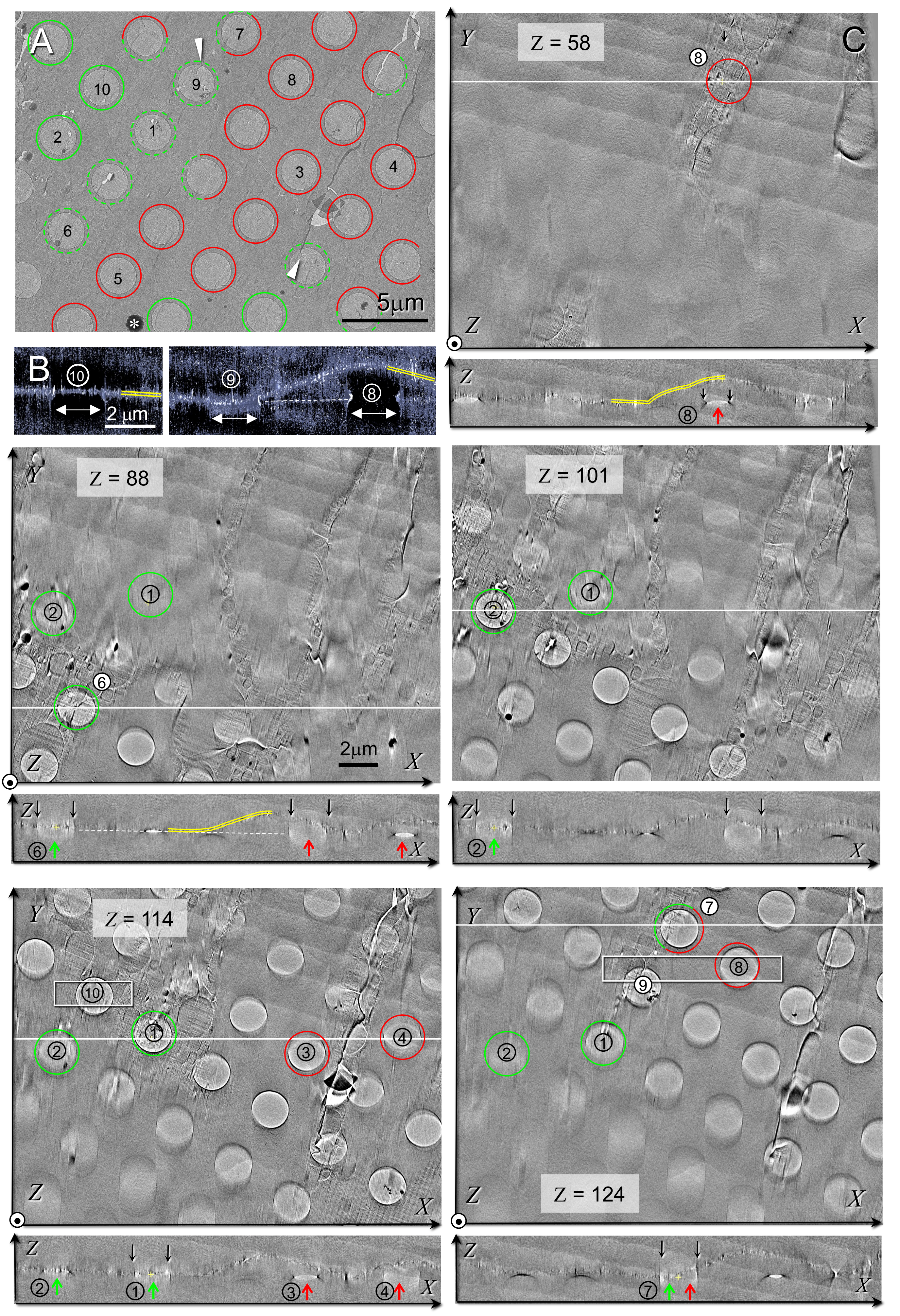

A
5μm
B
2 μm
C
Z = 58
Z = 88
2μm
Z = 101
Z = 114
Z = 124

Supplementary Figure S6

Figure S6. Adhesion mapping on atlases. 75 nm-thick section deposited on Quantifoil R2/1 reinforced by 30 nm carbon. 2-step charging procedure.
(Left) Two local views of an atlas recorded with a ~2 mm defocus. (Right) Adhesion map drawn from the differential contrast between adhesive (green overlay) and non-adhesive (red overlay) regions. A series of high magnification tomograms were recorded within squared regions. The colour code indicates the quality of their reconstructions.

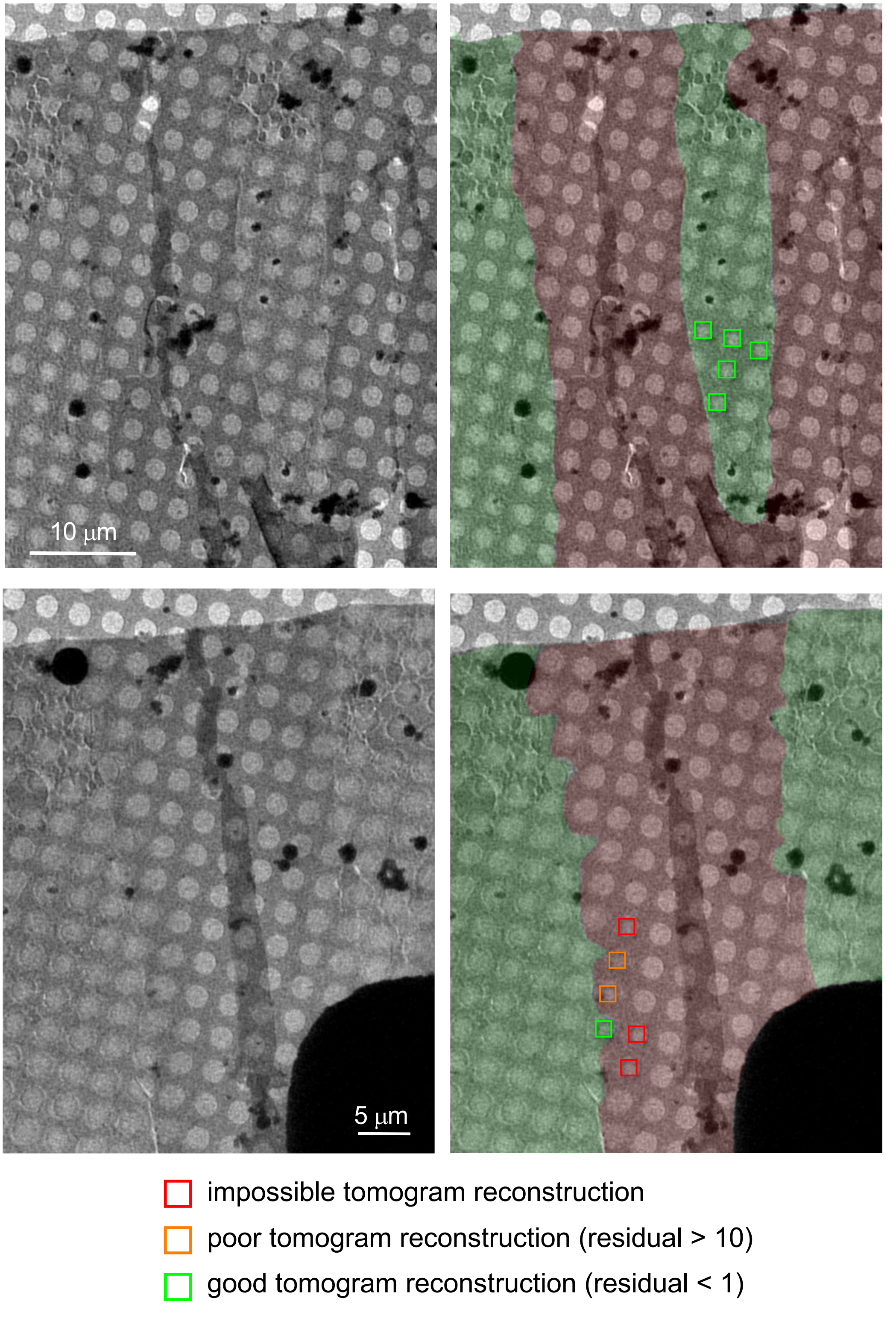

Supplementary Figure S7

Figure S7. Gallery of « lens » differential contrast observed in atlases and corresponding adhesion mapping sketch after 1-step (A) and 2-step (B) charging. Adhesive regions are coloured in green and non-adhesive ones in red.
Atlases were acquired with a defocus of ~ 0.1 to 0.15 mm (A1, A2) or 2 to 5 mm (A3, B).
In the examples shown here, 75 nm-thick sections are deposited on Quantifoil R2/2 (A1), R2/1 (A3, B2, B3) or S7/2 covered with ultralthin continuous carbon film (A2, B1).

A3 and A3' show two atlases of the same region (framed in the white rectangle in A3) respectively recorded at 300 kV in a Titan Krios G4 (Themofisher) and at 200 kV in a Glacios (Thermofisher). The same differential contrast between adhesive/non adhesive regions is observed. Note that contamination by ice crystals deposited on the sample surface has increased upon tranfer from Krios to Glacios (white asterisks).

A

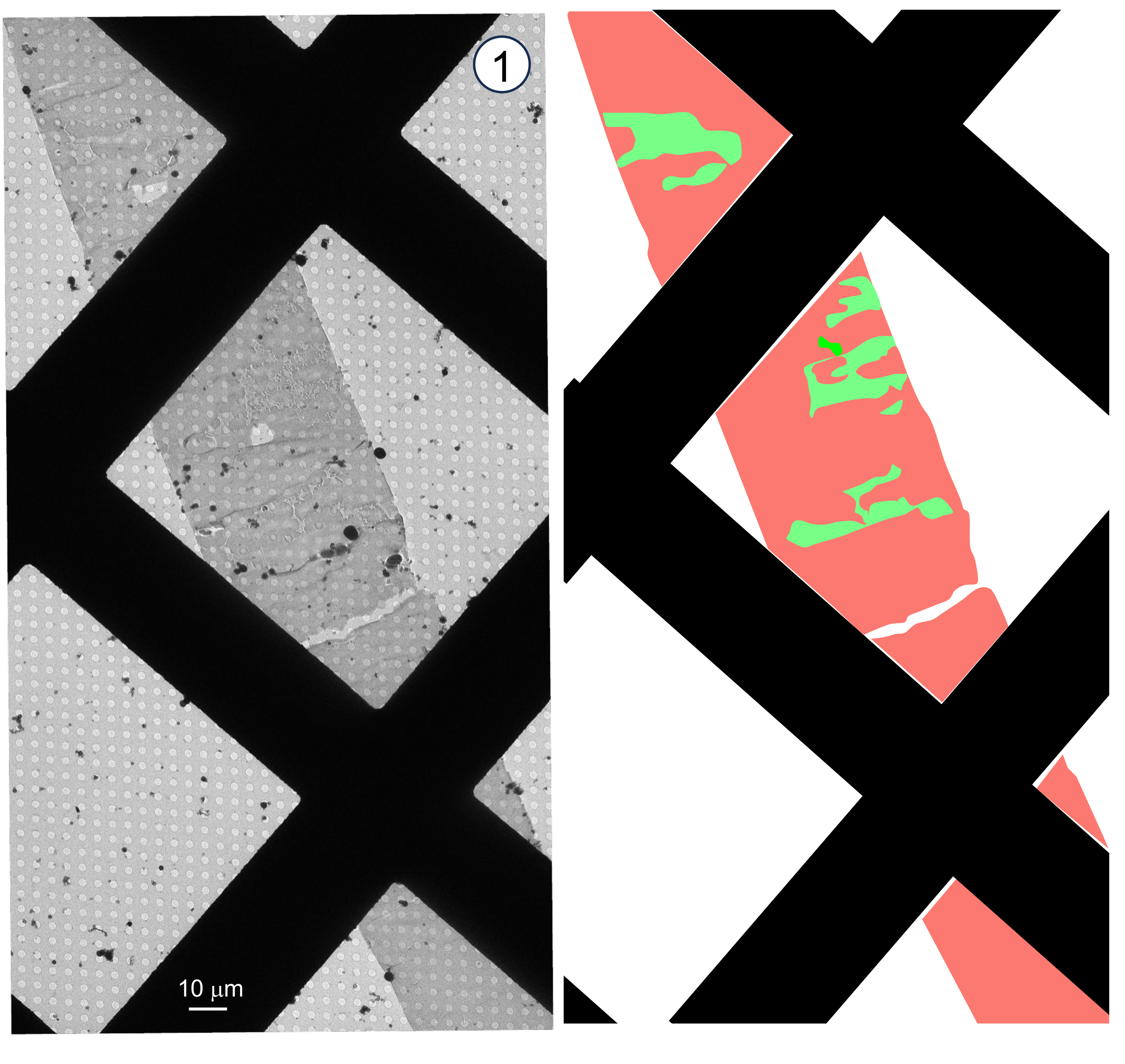

A

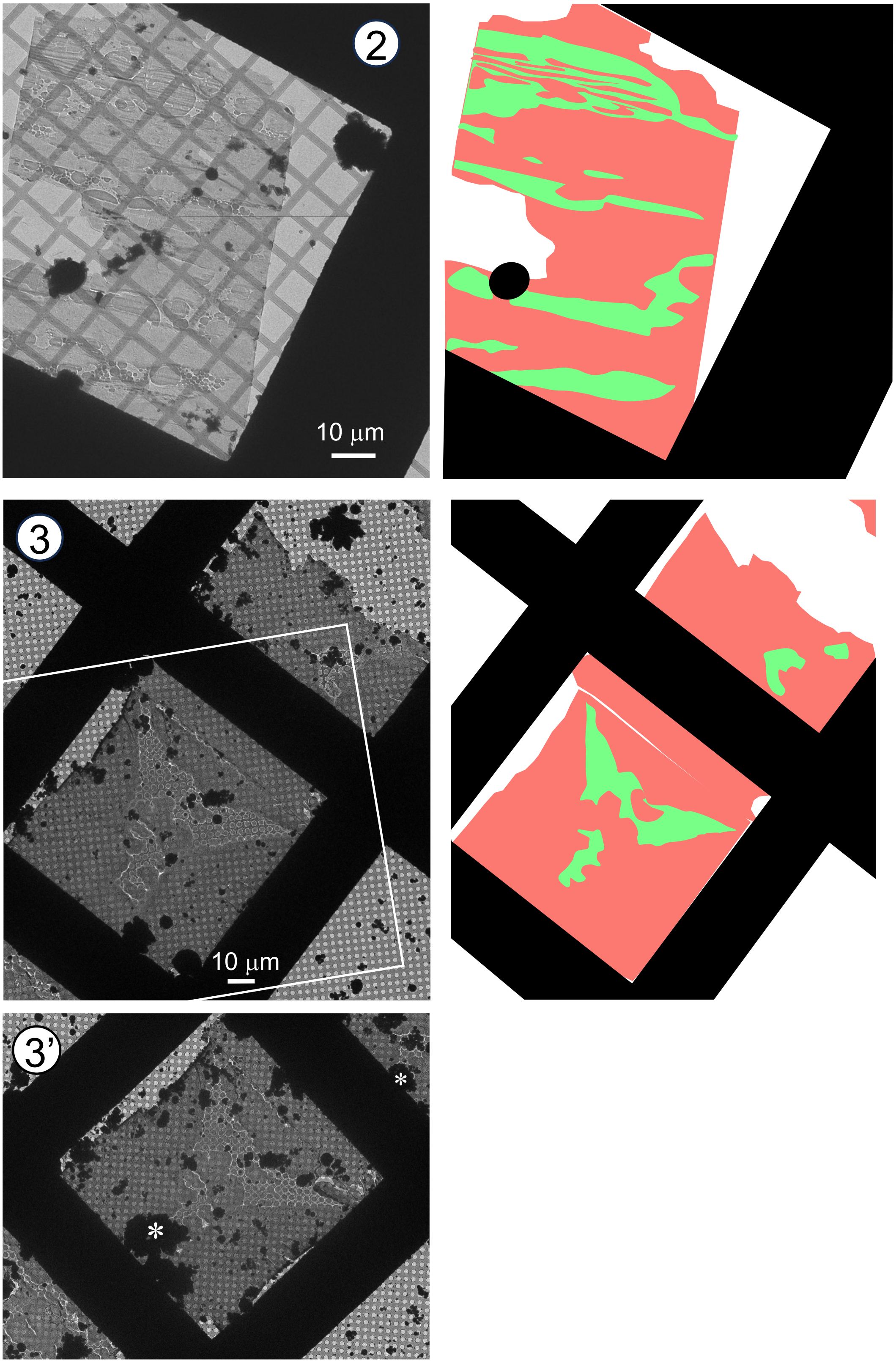

B

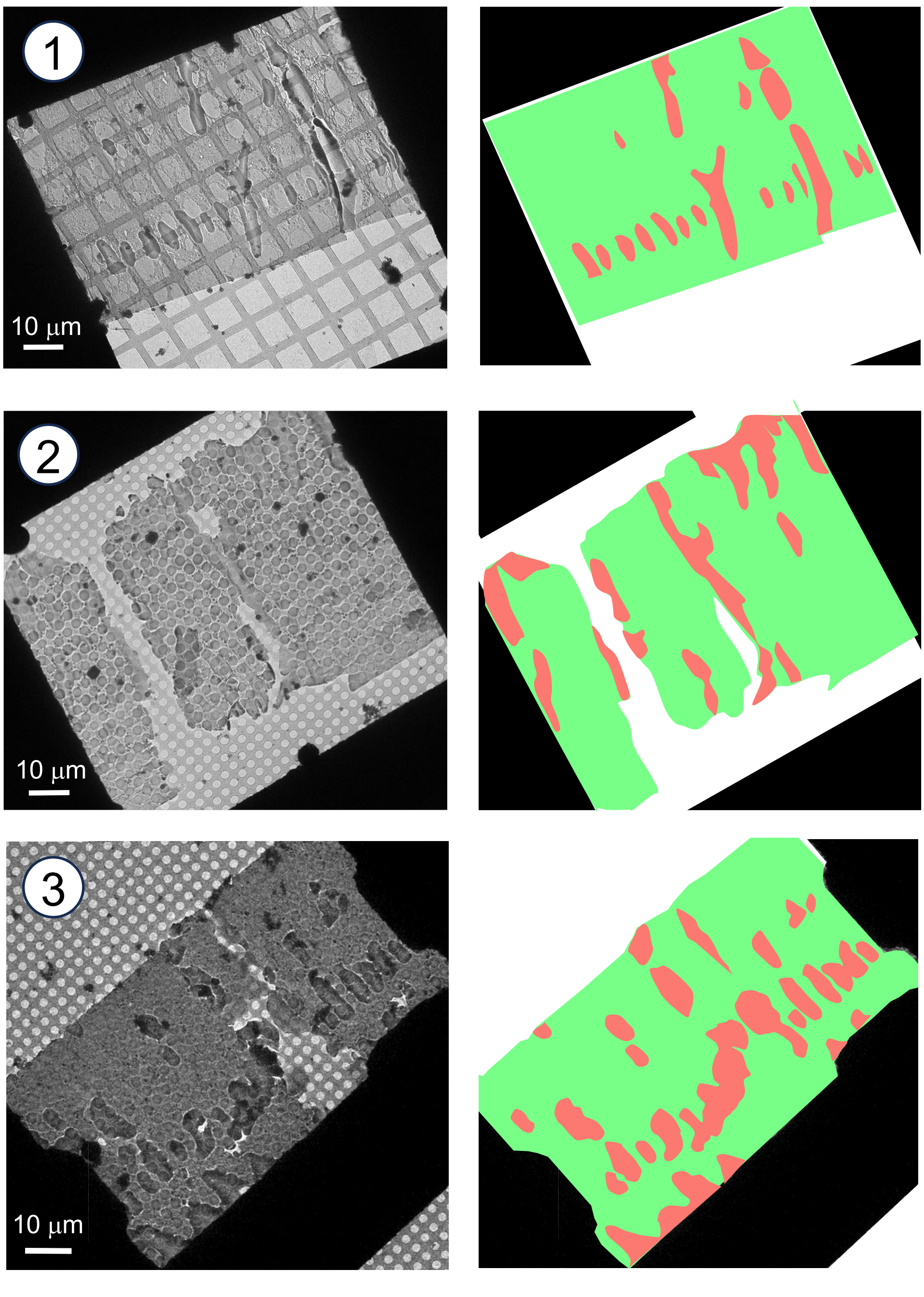

Supplementary Figure S8

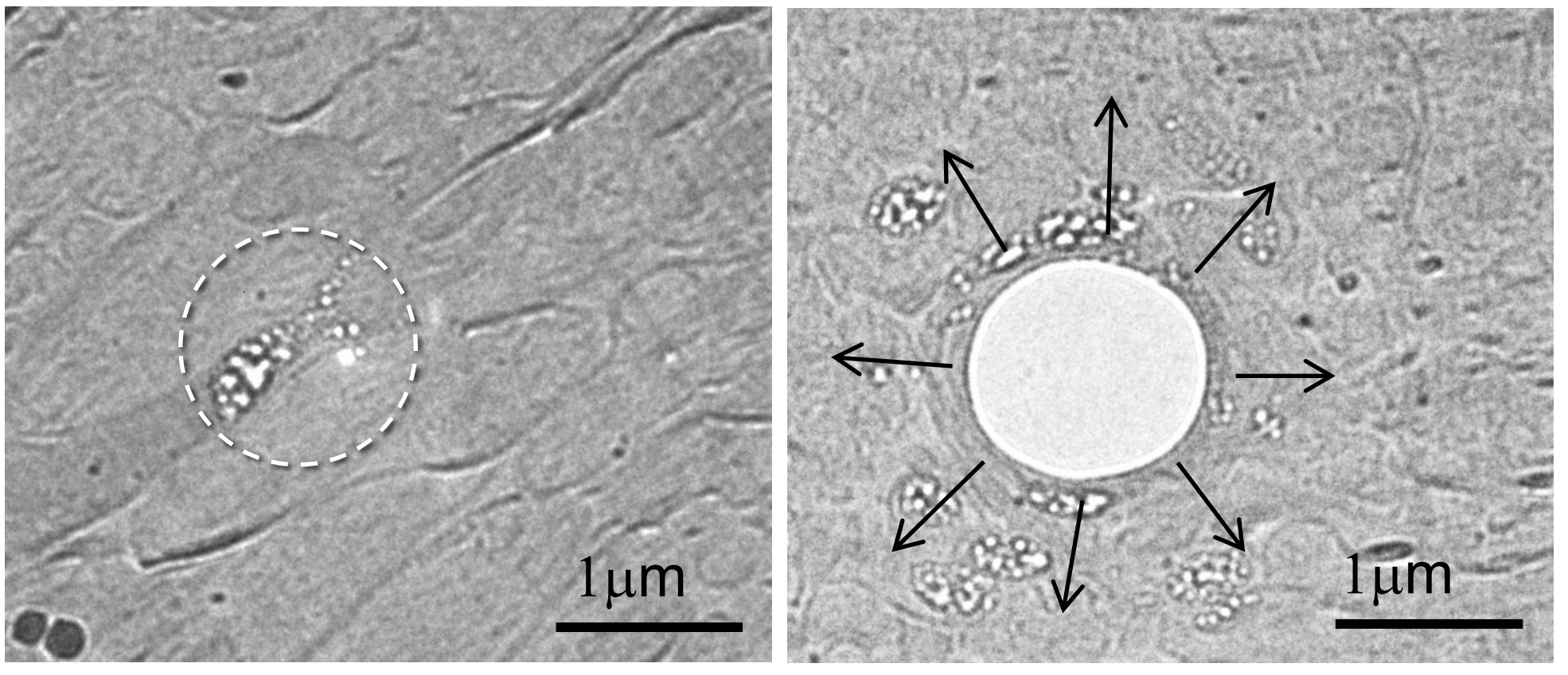

Figure S8. Focusing of the beam on an adhesive (left) and a non adhesive (right) region. Support grid: Quantifoil S7/2 + 2 nm continuous carbon film.

Supplementary Figure S9

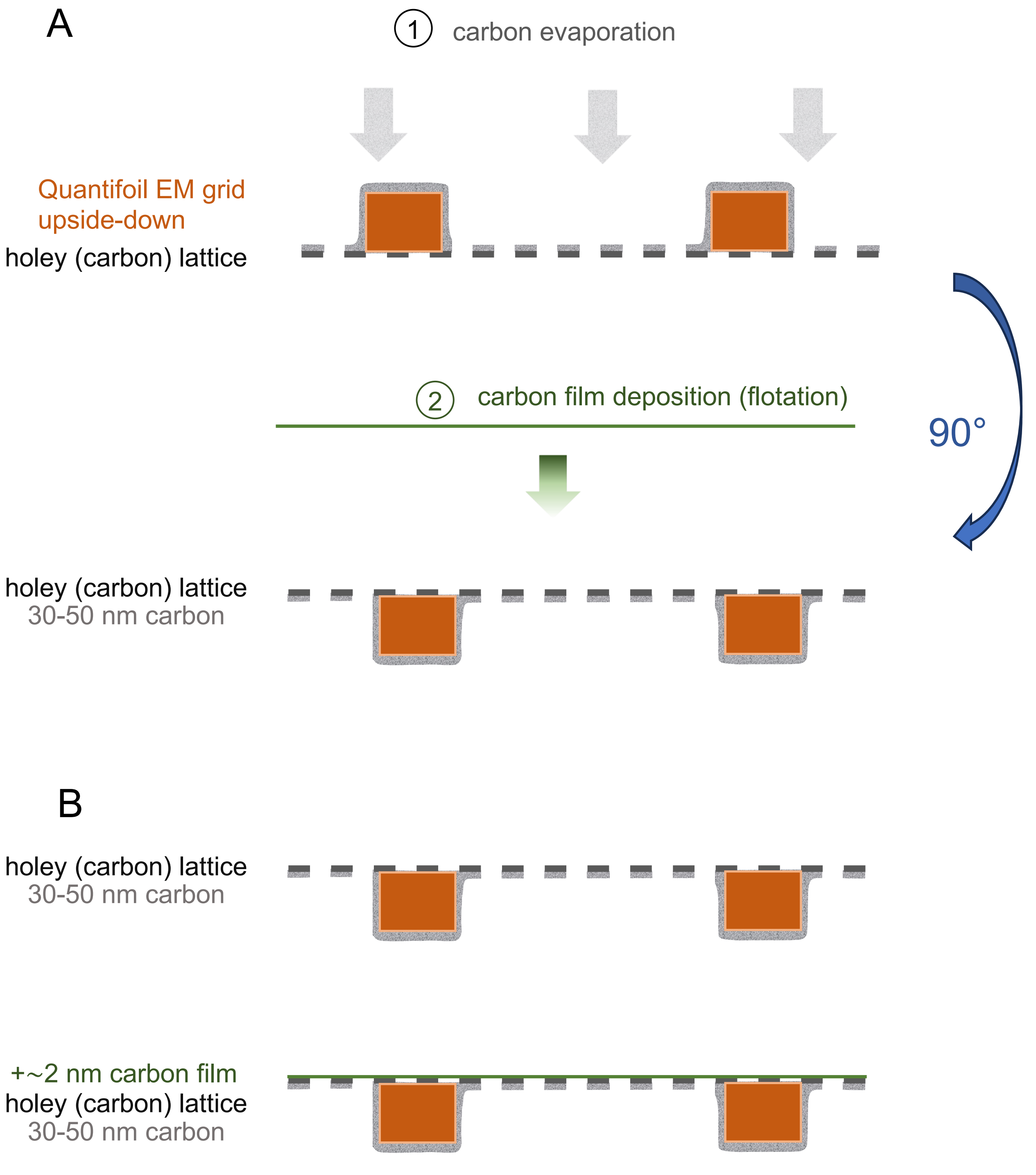

Figure S9. (A) Support film preparation, with strengthening of commercial support films by evaporation of an extra layer of carbon (1). On part of the grids, a continuous ultrathin carbon is then deposited by flotation (2). (B) Sketch of the two types of resulting support films.

Supplementary Figure S10

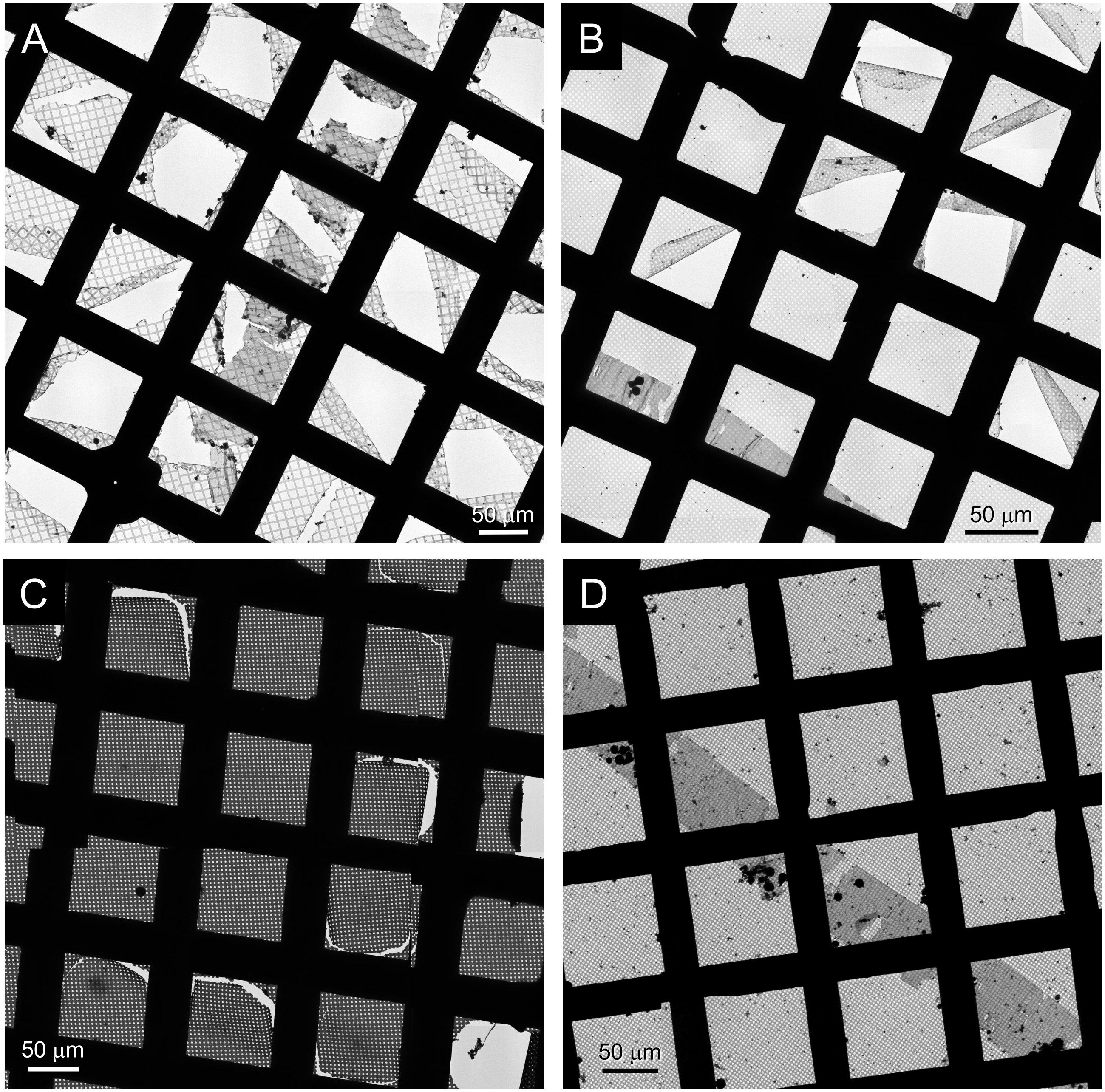

Figure S10. Representative examples of support grids after clipping, in the absence (A-C) or with (D) reinforcement with a 30 nm thick carbon layer. (A) Ribbon of cryosection deposited on Quantifoil S7/2 covered with an ultrathin carbon films (support 1 in Figure 4). (B) Ribbon of cryosection deposited on Quantifoil R2/2 (support 7 in Figure 4). (C) UltrAuFoil R2/2 (support 8). (D) Quantifoil R2/1 reinforced with a 30 nm carbon layer (support 6).
